# In vivo CRISPR screens reveal a HIF-1α-mTOR-network regulates T follicular helper versus Th1 cells

**DOI:** 10.1038/s41467-022-28378-6

**Published:** 2022-02-10

**Authors:** Bonnie Huang, James D. Phelan, Silvia Preite, Julio Gomez-Rodriguez, Kristoffer H. Johansen, Hirofumi Shibata, Arthur L. Shaffer, Qin Xu, Brendan Jeffrey, Martha Kirby, Stacie Anderson, Yandan Yang, Selamawit Gossa, Dorian B. McGavern, Louis M. Staudt, Pamela L. Schwartzberg

**Affiliations:** 1 National Institute of Allergy and Infectious Diseases, National Institutes of Health, Bethesda, MD 20892 USA; 2 National Human Genome Research Institute, National Institutes of Health, Bethesda, MD 20892 USA; 3 National Cancer Institute, National Institutes of Health, Bethesda, MD 20892 USA; 4 National Institute of Neurological Disorders and Stroke, National Institutes of Health, Bethesda, MD 20892 USA; 5 Present Address: TCR2 Therapeutics, Cambridge, MA 02142 USA

**Keywords:** CRISPR-Cas9 genome editing, Germinal centres, CD4-positive T cells, Systems analysis

## Abstract

T follicular helper (Tfh) cells provide signals to initiate and maintain the germinal center (GC) reaction and are crucial for the generation of robust, long-lived antibody responses, but how the GC microenvironment affects Tfh cells is not well understood. Here we develop an in vivo T cell-intrinsic CRISPR-knockout screen to evaluate Tfh and Th1 cells in an acute viral infection model to identify regulators of Tfh cells in their physiological setting. Using a screen of druggable-targets, alongside genetic, transcriptomic and cellular analyses, we identify a function of HIF-1α in suppressing mTORC1-mediated and Myc-related pathways, and provide evidence that VHL-mediated degradation of HIF-1α is required for Tfh development; an expanded in vivo CRISPR screen reveals multiple components of these pathways that regulate Tfh versus Th1 cells, including signaling molecules, cell-cycle regulators, nutrient transporters, metabolic enzymes and autophagy mediators. Collectively, our data serve as a resource for studying Tfh versus Th1 decisions, and implicate the VHL-HIF-1α axis in fine-tuning Tfh generation.

## Abstract (web summary)

T follicular helper (Tfh) and T help type 1 (Th1) cells both arise from naïve CD4 T cells, but detailed knowledge of their differentiation remains incomplete. Here the authors pursue an in vivo CRISPR screen to identify genes, focusing on druggable targets, regulating Tfh versus Th1 to provide a resource for related studies, while also implicating HIF-1α and VHL in this regulation.

## Introduction

T follicular helper (Tfh) cells provide critical signals for the formation and maintenance of germinal centers (GCs), the sites of immunoglobulin gene somatic hypermutation, antibody affinity maturation, and differentiation of long-lived plasmablasts and memory B cells^1^. The generation of Tfh cells has been correlated with robust vaccine responses, including those to SARS-CoV-2, while an excess of Tfh cells is found in many autoimmune diseases, supporting an important role for Tfh cells as a regulatory arm of humoral immune responses^1^. Past studies have identified transcription factors that promote Tfh differentiation, including BCL-6, ASCL2, TCF-1, and STAT3, along with others that oppose Tfh development or promote alternative Th fates, including BLIMP-1, STAT5, FOXO1, and STAT1^1^. Beyond these molecular control elements, the physical positioning of Tfh cells within follicles and GCs is also crucial for Tfh differentiation and function, as it enables their interactions with B cells. Spatial localization and movement of Tfh are regulated by a multitude of chemokine and adhesion receptors including CXCR5, CCR7, S1PR1/2, EBI2, LFA-1, and SLAM family members^2^. However, our understanding of Tfh physiology remains incomplete. Increasingly, it is becoming clear that Tfh differentiation is also determined by GC microenvironmental factors, such as the metabolic fuels glucose, glutamine, and fatty acids^3–6^, which may be limiting resources for which GC cells compete^7,8^, as well as immunosuppressive molecules ATP^9^ and adenosine^10^, which may be released by other GC populations. How these influence the balance of Tfh cells and other CD4^+^ effector T cell populations is less appreciated.

Here, we use CRISPR gene targeting to screen both primary immunodeficiency (PID) genes, as well as the druggable genome expressed in Tfh cells, for roles in Tfh versus Th1 differentiation during acute viral infection. We uncover a role for hypoxia-inducible factor-1 (HIF-1α) in repressing Tfh cells, and show that loss of VHL, the Von-Hippel-Lindau E3-ubiquitin ligase that targets HIF-1α for degradation, inhibits Tfh generation and dampens mechanistic Target of Rapamycin Complex1 (mTORC1) activity. Nonetheless, although increasing mTORC1 activation rescues the balance of Tfh:Th1 cells in the absence of VHL, this fails to rescue defects in cell expansion associated with decreased Myc. An expanded in vivo CRISPR screen highlights opposing effects of HIF-1α and mTOR pathways while serving as a resource of multiple players affecting Tfh and Th1 cells. Our results suggest that T cells use HIF-1α as a gauge of the GC environment to tune both mTOR activation and proliferation, and thereby regulate Tfh cells and GC responses.

## Results

### Efficient CRISPR-gene targeting in primary murine T cells

**Fig. 1:**
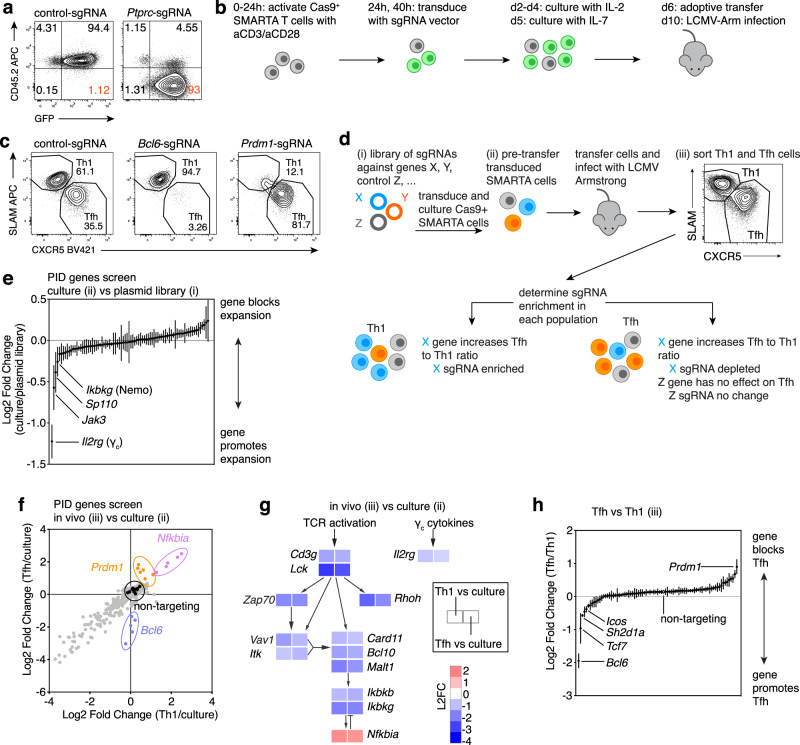
CRISPR-mediate gene knockout in primary mouse T cells and screen of PID genes. **a** CD45 on Cas9^+^ CD4 T cells transduced with MRIG-control or -*Ptprc*-sgRNA at d6 in culture. **b** Schematic of Cas9^+^ SMARTA cells transduction, adoptive transfer into WT hosts, and LCMV Armstrong infection. Green:transduced cells. **c** Representative flow plots of Tfh:Th1 differentiation of Cas9^+^ SMARTA cells transduced with control-, *Bcl6*-, or *Prdm1*-sgRNA, d6 post-LCMV. **d** In vivo screening schematic, indicating samples collected for DNA and quantification of sgRNA-sequences by deep sequencing from: (i) pooled sgRNA library, (ii) cultured transduced Cas9^+^ SMARTA T cells, and (iii) isolated Th1 and Tfh populations after adoptive transfer and infection with LCMV Armstrong. Blue:gene required for Tfh; Orange:gene required for Th1 cells; Grey:no effect on Tfh cells **e** Log2 fold change (L2FC) of sgRNA relative abundance, comparing Cas9^+^ SMARTA cells at d6 culture (prior to adoptive transfer) to PID sgRNA plasmid library. Each symbol represents mean of all sgRNAs for one gene + /-SEM. **f**–**h** Cas9^+^ SMARTA cells transduced with PID library were sorted into Th1 and Tfh populations on d6 post-LCMV infection. **f** L2FC of sgRNA relative abundance, comparing Tfh/culture versus Th1/culture. Each symbol represents an individual sgRNA. Colors as in (**d**) plus black:controls; purple:gene inhibits cell-expansion; grey:gene required for cell-expansion. **g** L2FC of Tfh/culture and Th1/culture for sgRNAs targeting select genes involved in TCR and NF-κB signaling. L2FC are means of all sgRNAs for each gene. **h** L2FC of sgRNA relative abundance comparing Tfh versus Th1. Each symbol represents the mean of all sgRNAs for one gene + /-SEM. **e**–**h** Data pooled from 2 independent experiments. *n* = 9 mice total (first experiment *n* = 4, second experiment *n* = 5). Cells from each mouse were sorted individually. Ratio and L2FC values were calculated as the mean for each mouse for each experiment, then averaged across experiments. Source data provided in Source Data file and Supplementary Data 3.

To disrupt genes in primary murine T lymphocytes, we generated two sets of retroviral vectors^11^ that expressed a single guide RNA (sgRNA) driven by the mouse U6 promoter. One vector included the Cas9 nuclease from *S. pyogenes* and a GFP marker (MRCIG), whereas a smaller vector (MRIG) contained only the U6-sgRNA and GFP (Supplementary Fig. 1a)^12^ and was used to transduce T cells from transgenic Cas9-expressing mice^13^. Primary CD4^+^ T cells were activated and transduced with these vectors containing sgRNAs directed against the genes encoding Thy1 or CD45 (*Ptprc*). Using the larger vector, we found a fraction of the GFP^+^ population had lost Thy1 or CD45 after 6 days in culture (Supplementary Fig. 1b, c) and could detect distinct patterns of indel mutations in the targeted genes using a rapid fluorescent PCR-based method^14^ (Supplementary Fig. 1d, e). However, transduction and gene knockout rates were modest, likely because the vector size approached the packaging limits of murine retroviruses. In contrast, transduction of Cas9-expressing T cells with the smaller vector yielded >90% GFP^+^ cells, almost all of which lost the targeted protein (Fig. 1a, Supplementary Fig. 1f).

### CRISPR-based modulation of in vivo Tfh differentiation

To evaluate the effects of CRISPR-mediated gene knockout on Tfh differentiation, we used an acute viral infection model^15^ and adoptive transfer of T cells from SMARTA mice, which express a transgenic TCR directed against a dominant glycoprotein epitope of lymphocytic choriomeningitis virus (LCMV) presented by MHC-II^16^. Activated CD4^+^ T cells from Cas9^+^ SMARTA mice were transduced with MRIG containing control non-targeting sgRNAs, or sgRNAs directed against *Bcl6* or *Prdm1* (encoding Blimp1), which encode key transcription factors that promote and repress Tfh differentiation, respectively^1^, then adoptively transferred into WT recipients, which were subsequently infected with LCMV Armstrong (Fig. 1b)^12,17^. Under these conditions, WT SMARTA transgenic cells rapidly expand and differentiate into two major populations: CXCR5^+^SLAM^−^ Tfh cells and CXCR5^−^SLAM^+^ Th1 cells^18^. In contrast, *Bcl6*-sgRNA-targeted SMARTA cells developed few Tfh cells at day 6 post-LCMV infection (p.i.), whereas *Prdm1*-sgRNA SMARTA exhibited significantly decreased Th1 cells, with increased percentages and numbers of Tfh cells (Fig. 1c, Supplementary Fig. 1g, h). SgRNA targeting of *Tcf7*, which encodes TCF-1, another transcription factor important for Tfh cell generation in response to viral infection^19^, led to loss of TCF-1 protein and reduced Tfh cell generation (Supplementary Fig. 1i, j). Thus, gene-targeted T cells could be transferred back into mice and evaluated for differentiation in vivo.

To test the sensitivity of this system for multiplexed screening^15^, different numbers of *Tcf7-*sgRNA-transduced SMARTA T cells were spiked into a total of 1 × 10^6^ mock-transduced SMARTA CD4^+^ T cells, the upper limit of transferred antigen-specific cells that permits appropriate responses to LCMV^15^. Postinfection, GFP^+^ sgRNA-transduced were compared to GFP^-^ mock-transduced SMARTA cells within each host. Tfh defects could be detected at a 1 in 1000 frequency of *Tcf7*-sgRNA cells, which was equivalent to 1000 transferred targeted cells (Supplementary Fig. 1k). Since adoptive transfer results in about 90% loss of T cells, this represents the transfer of about 100 targeted cells, a number similar to precursor frequencies of antigen-specific T cells^20^. Thus, cell-autonomous effects on the differentiation of sgRNA-transduced SMARTA cells could be detected within a pool of about 1000 sgRNAs and still allow functional readout of each sgRNA.

### CRISPR-based functional genetic screening of PID genes

As a proof-of-concept screen for genes with putative roles in CD4 T cell responses to LCMV, we first constructed a pooled retroviral sgRNA library targeting ~80 genes associated with PIDs^21^. Non-targeting negative control sgRNAs and positive control sgRNAs affecting Tfh differentiation were included in a pool of approximately 400 sgRNAs (Supplementary Data 2). Genomic DNA was isolated and sgRNA sequences were PCR amplified and quantified by next-generation sequencing at three stages: (i) the plasmid library, (ii) pretransfer in vitro cultured transduced SMARTA cells, and (iii) Th1 and Tfh SMARTA populations isolated by cell sorting based on CXCR5 and SLAM expression 6 days p.i. (Fig. 1d, Supplementary Data 3).

To detect whether genes impaired SMARTA expansion in vitro, we compared relative abundances of each sgRNA at the end of in vitro culture to that of the plasmid library. The most depleted sgRNAs were against *Il2rg* (γ_c_) and *Jak3* (Fig. 1e), consistent with the use of IL-2 during in vitro expansion of transduced T cells. We then compared relative frequencies of each sgRNA in sorted Th1 or Tfh cells to levels in culture prior to transfer. Many sgRNAs were depleted from both Th1 and Tfh populations (Fig. 1f, bottom left), suggesting that these PID genes were important for both cell lineages and/or overall expansion in response to LCMV; these included multiple genes involved in T cell receptor signaling and NF-κB activation (Fig. 1g), suggesting a critical role for this pathway in T cell expansion after LCMV infection. In contrast, sgRNAs directed against *Nfkbia*, encoding an NF-κB inhibitor IκBα, were enriched after LCMV infection (Fig. 1f, g).

We then compared sgRNA abundance in sorted Tfh versus Th1 cells (Fig. 1h, Supplementary Fig. 2a). Comparison of these populations allowed us to evaluate their relative generation, independent of effects on SMARTA T cell expansion in vivo. The most depleted sgRNAs in Tfh cells included those directed against *Bcl6*, while the most enriched were against *Prdm1* (Fig. 1h and Supplementary Fig. 2a); non-targeting control sgRNAs were not enriched in either population. SgRNAs against the genes *Icos*^18^ and *Sh2d1a*^22^, which are associated with genetic defects in Tfh cells and humoral responses, as well as *Stat1*, *Rhoh*, and *NfkB2* were all depleted in Tfh cells. Furthermore, multiple sgRNAs, including those directed against *Il2rg* and *Jak3* were enriched in Tfh cells, supporting roles for IL-2 signaling in repressing Tfh and/or promoting Th1 cells. Thus, we were able to confirm the importance of several known PID genes and detect others that affect Tfh cell differentiation.

### Druggable-target screen reveals roles for HIF-1α in Tfh cells

To screen genes with potentially unknown function in Tfh differentiation, we manually curated a list of genes from the Druggable Genome database^23^ that were transcriptionally expressed in Tfh cells and used these to construct a retroviral library of approximately 400 sgRNAs (see Methods and Supplementary Data 4, 5). To refine our screen and detect candidate genes that differentially affect pre-Tfh (CXCR5^+^PD-1^+^, which also include central memory precursors^24^) and GC Tfh (CXCR5^hi^PD-1^hi^) cells, we altered our sorting strategy to isolate these populations separately in addition to Th1 (CXCR5^−^PD-1^+^) cells (Supplementary Fig. 2b). Screen results were highly similar between pre-Tfh-versus-Th1 and GC Tfh-versus-Th1 cells (Supplementary Fig. 2c, left), and from spleen versus peripheral lymph nodes (Supplementary Fig. 2c, middle), consistent with this being a systemic infection. Screen results were also similar on day 6 versus 7 p.i. (Supplementary Fig. 2c, right), both of which are within the timeframe of peak primary T cell responses to LCMV infection.

**Fig. 2:**
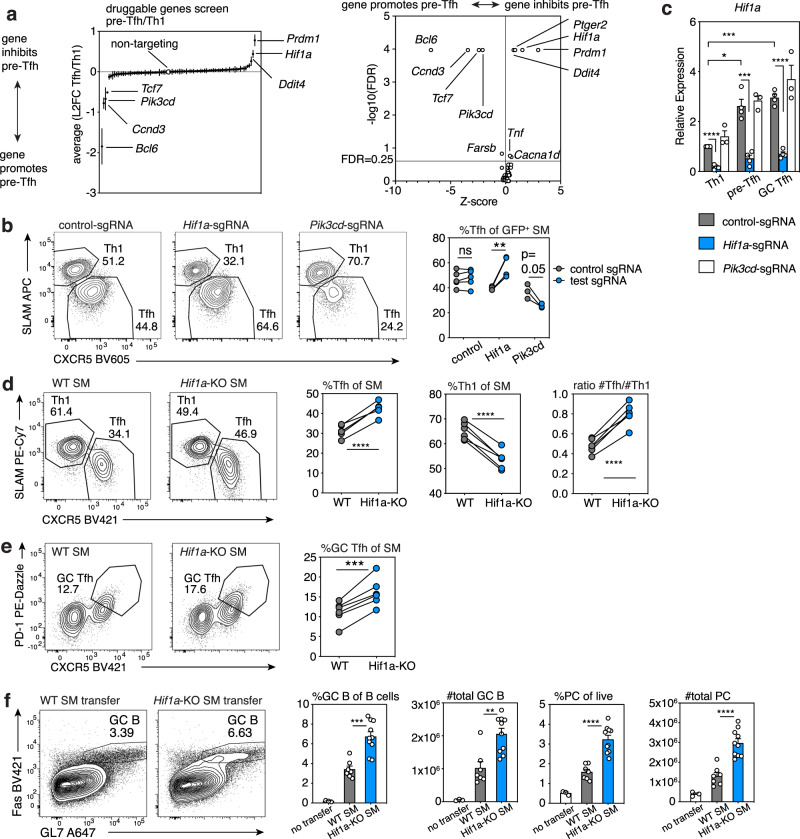
Druggable-target CRISPR screen reveals disparate roles for PI3K p110δ and HIF-1α. **a** Druggable targets sgRNA screen (single experiment, sorted as in Supplementary Fig. 2a post-LCMV, *n* = 17 mice (d6 *n* = 8; d7 *n* = 9). Left: L2FC of sgRNA pre-Tfh versus Th1 relative abundance. Each symbol represents mean of all sgRNAs for one gene + /-SEM. Right: Screen results analyzed by Mageck for FDR and L2FC to generate Z-scores. Genes with FDR < 0.25 are indicated. **b** Representative flow plots of Tfh:Th1 differentiation in WT hosts receiving co-transferred CD45.2/.2 Cas9^+^ control-sgRNA-transduced SMARTA cells and CD45.1/.2 Cas9^+^ SMARTA cells transduced with indicated sgRNA vectors, analyzed d6 post-LCMV. Control-sgRNA *n* = 5, *Hif1a*-sgRNA *n* = 4, *Pik3cd*-sgRNA *n* = 3. **c** qRT-PCR of *Hif1a* mRNA in Cas9^+^ SMARTA cells transduced with indicated MRIG sgRNA vectors, transferred into WT hosts, and sorted d6 p.i. Data are pooled from *N* = 3–4 independent experiments (control-sgRNA and *Hif1a*-sgRNA *N* = 4; *Pik3cd*-sgRNA *N* = 3) using 3–8 mice/genotype/experiment. Within each experiment, relative expression was normalized to control-sgRNA Th1 cells. **d** Tfh:Th1 representative flow plots in CD45.1/.2 hosts receiving co-transferred naïve CD45.1/.1 WT and CD45.2/.2 *Hif1a*-KO-SMARTA cells, on d8 post-LCMV, *n* = 6 mice/group. **e** GC Tfh representative flow plots and percentages for experiment in (**d**). **f** Representative flow plots and frequencies and total numbers of GC (Fas^+^GL-7^+^) B cells among CD19^+^B220^+^ B cells, plasma cell (B220^med^CD138^+^) (PC) among live cells, in *Bcl6*^fl/fl^*Cd4*-Cre^+^ hosts that received no T cells, naïve WT or naïve *Hif1a*-KO SMARTA cells, analyzed d8 post-LCMV infection. WT SMARTA recipients *n* = 7, *Hif1a*-KO SMARTA recipients *n* = 10, controls *n* = 3. Data in (c) and (f) represent mean +SEM. Representative data (**b**), (**d**–**f**) shown from 1 of at least 2 independent experiments. *p < 0.05; **p < 0.01, ***p < 0.001, ****p < 0.0001, evaluated by two-tailed unpaired (**c**, **f**) or paired (**b**, **d**, **e**) Student’s *t* test. Source data provided in Source Data file and Supplementary Data 5.

In addition to *Bcl6* and *Tcf7*, the other most depleted sgRNAs in pre-Tfh cells targeted Phosphoinositide 3-Kinase (PI3K) p110δ (*Pik3cd*) and the cell cycle regulator, Cyclin D3 (*Ccnd3*) (Fig. 2a). Previous reports have demonstrated important roles for p110δ in Tfh differentiation^25,26^. However, a role for Cyclin D3 in Tfh cells has not previously been appreciated, although Cyclin D3 is required to drive proliferation of GC B cells^27,28^.

In contrast, sgRNAs directed against multiple genes, including *Hif1a*, *Ddit4* (a HIF-1α downstream target), and *Ptger2* (encoding prostaglandin receptor E2) were increased in abundance in pre-Tfh cells, suggesting these genes skewed the ratio of Tfh:Th1 cells away from Tfh (Fig. 2a). The finding of *Hif1a* was surprising, since in many cell types, PI3K can activate mTORC1^29^, which, in turn, induces *Hif1a* expression^30,31^. As both p110δ and mTOR are required for full Tfh differentiation^3,25,26,32–34^, loss of HIF-1α might be expected to impair Tfh differentiation. Furthermore, two reports supported a role for HIF-1α in promoting Tfh cells when HIF-1α was deleted in all T cells^35,36^. Targeting *Pik3cd* with an individual sgRNA reduced both Tfh percentages and numbers compared to control-sgRNA-treated cells co-transferred to the same hosts, so that cells were exposed to similar viral titers and environment (Fig. 2b, Supplementary Fig. 2d). In contrast, *Hif1a*-sgRNA increased percentages of Tfh cells relative to Th1 cells (Fig. 2b), although absolute numbers varied due to variable effects on cell expansion (Supplementary Fig. 2e). These findings did not result from altered ratios of T follicular regulatory (Tfr) to Tfh cells, because SMARTA cells generate very few Tfr cells after adoptive transfer and infection (Supplementary Fig. 2f). Evaluation of mRNA by qRT-PCR confirmed that *Hif1a* mRNA was higher in SMARTA pre-Tfh and GC Tfh than in Th1 cells and was lost in all three populations in *Hif1a*-sgRNA-treated cells (Fig. 2c). In contrast, *Hif1a* mRNA was comparable in *Pik3cd*-sgRNA and control-sgRNA-treated SMARTA populations, suggesting that loss of PI3Kδ does not affect *Hif1a* expression in this context.

To confirm these findings, we crossed *Hif1a*^*fl/fl*^ mice to *Cd4-Cre* mice to selectively delete *Hif1a* in T cells. Comparison of Cre^−^ versus Cre^+^ mice at baseline, (designated WT and *Hif1a*-KO, respectively) showed *Hif1a*-KO mice had similar percentages and numbers of endogenous Tfh, Tfr, and GC B (Supplementary Fig. 3a). However, co-adoptive transfer of naïve WT and *Hif1a*-KO SMARTA cells into WT hosts, which were then infected with LCMV, confirmed increased Tfh and GC Tfh and decreased Th1 frequencies in the latter, as evaluated by either CXCR5/SLAM or CXCR5/PD-1 staining (Fig. 2d, e). Thus, *Hif1a*-KO Tfh cells were increased compared to WT Tfh cells in the same animal (Supplementary Fig. 3b). Postinfection, *Hif1a*-KO SMARTA cells expressed similar levels of BCL-6 and TCF-1, but lower T-bet and slightly higher ICOS than WT (Supplementary Fig. 3c). Expression of IL-21 and surface-CD40L were also similar to WT upon in vitro restimulation (Supplementary Fig. 3d). Increased Tfh and decreased Th1 frequencies in *Hif1a*-KO SMARTA cells were also observed at day 3 p.i. (a timepoint used to evaluate early differentiation and expansion), as indicated by staining with TIM-3 versus CXCR5 which demarcate early Th1 and Tfh cells^19^ (Supplementary Fig. 3e).

*Bcl6*^*fl/fl*^*;Cd4-Cre* mice cannot form Tfh cells and therefore fail to generate GCs. When transferred into *Bcl6*^*fl/fl*^*;Cd4-Cre* hosts, *Hif1a*-KO SMARTA cells rescued GC and isotype switched B cell and plasma cell numbers to a greater extent than WT SMARTA cells in response to LCMV (Fig. 2f and Supplementary Fig. 3f). Thus, *Hif1a*-deficiency increased the generation of functional Tfh cells that can provide help for GC formation. Nonetheless, GC B cells that developed in response to *Hif1a*-KO T cells exhibited slightly higher ratios of the dark zone (DZ) to light zone (LZ) partitioning, as evaluated by CXCR4 and CD86 staining (Supplementary Fig. 3f), possibly due to increased Tfh cells^37^.

### HIF-1α-dependent response to hypoxia alters Tfh:Th1 balance

**Fig. 3:**
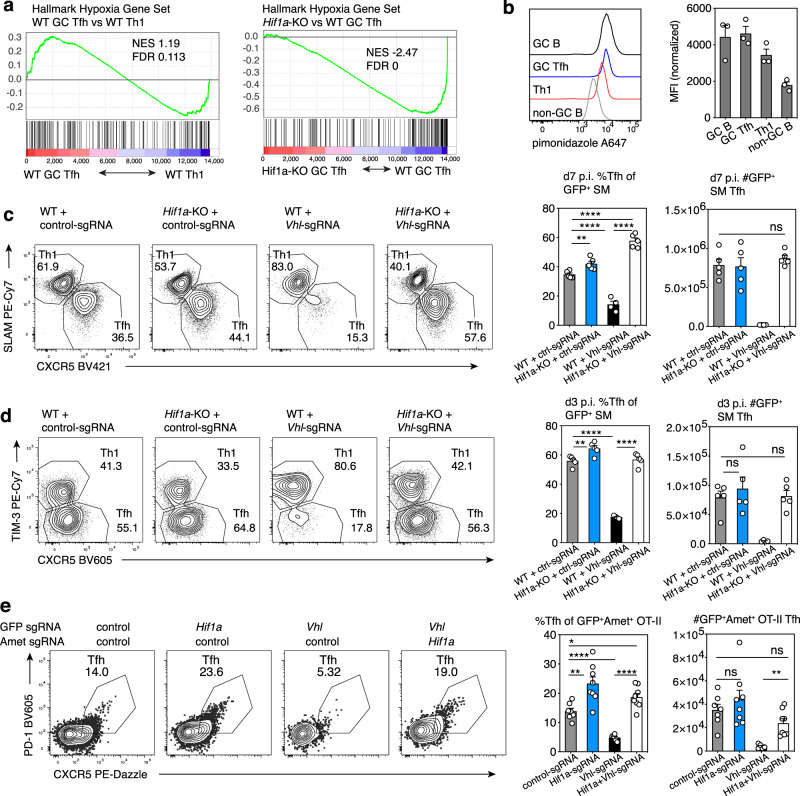
Hypoxia and loss of VHL repress Tfh differentiation. **a** GSEA analysis of “Hallmark Hypoxia” gene set in bulk RNA-seq data from WT or *Hif1a*-KO SMARTA cells transferred into WT hosts and sorted for Th1, pre-Tfh, and GC Tfh cell populations on d8 post-LCMV infection. NES, normalized enrichment score. **b** Pimonidazole staining of host B cells and WT SMARTA cells after transfer into WT hosts, on d8 post-LCMV infection. MFIs were normalized to cells from control hosts receiving WT SMARTA cells and saline injections, infected and processed in parallel. *n* = 3 pimonidazole-injected mice. **c**, **d** Representative flow plots, Tfh percentages and total Tfh cells from WT or *Hif1a*-KO Cas9^+^ GFP^+^ SMARTA cells transduced with control- or *Vhl*-sgRNA vector and transferred into WT hosts, on d6 (**c**) and d3 (**d**) post-LCMV infection, *Vhl*-sgRNA *n* = 4 mice, all other groups *n* = 5. **e** Representative flow plots, Tfh percentages and total Tfh cells from GFP^+^Ametrine^+^ Cas9^+^ OT-II cells after transduction with the indicated sgRNA vectors and transferred into WT hosts, on d6 post-immunization with NP-ovalbumin/alum, control-sgRNA *n* = 7, *Hif1a*-sgRNA *n* = 8, *Vhl*-sgRNA *n* = 5, *Hif1a*-sgRNA + *Vhl*-sgRNA *n* = 8. Data in (**b**–**e**) are presented as mean values +SEM. Representative data for (**b**–**e**) shown from 1 of at least 2 independent experiments. *p < 0.05; **p < 0.01, ***p < 0.001, ****p < 0.0001 as evaluated by two-tailed unpaired Student’s *t* test (**c**–**e**). Source data provided in Source Data file and Supplementary Data 6.

In addition to mTORC1-mediated induction of *Hif1a*, HIF-1α protein is regulated by hypoxia. Under oxygen-replete conditions, prolyl hydroxylases (PHDs) hydroxylate HIF-1α, targeting it for proteasomal degradation by the VHL E3-ubiquitin ligase. During hypoxia, PHDs are inactive, VHL is not recruited, and HIF-1α is stabilized^38^. Several recent reports suggest that GCs display features of a hypoxic microenvironment^39–41^, particularly the LZ^39,42^ where GC Tfh cells localize, although this remains controversial^43^. To evaluate this issue, we sorted WT and *Hif1a*-KO-transferred SMARTA Th1, Tfh, and GC Tfh cells and performed RNA-seq. GSEA revealed that GC-Tfh cells showed a mild enrichment for the MSigDB hallmark hypoxia gene signature (Fig. 3a); similar results were seen with published Tfh gene expression datasets (Supplementary Fig. 4a). This signature was HIF-1α-dependent, since its enrichment was lost when comparing *Hif1a*-KO GC Tfh to WT GC Tfh (Fig. 3a). In vivo treatment of LCMV-infected mice with a hypoxia probe, pimonidazole, revealed slightly higher labeling in SMARTA GC Tfh cells compared to Th1 cells in the same animals, similar to levels in GC B cells (Fig. 3b).

Increased Tfh cell percentages in the absence of HIF-1α suggested that elevated HIF-1α protein is detrimental for Tfh differentiation. However, absolute numbers were not consistently increased and it remained unclear whether the effects we observed resulted from decreased Th1 differentiation versus inhibitory effects on Tfh cells. To test this question, we used CRISPR to knockout *Vhl*, loss of which leads to constitutively high stable HIF-1α protein (Supplementary Fig. 4b)^38^. *Vhl*-deficiency markedly impaired SMARTA cell expansion (Supplementary Fig. 4c), However, *Vhl*-deficiency more severely impaired generation of Tfh than Th1 cells, resulting in decreased Tfh:Th1 ratios (Fig. 3c, Supplementary Fig. 4c). Loss of VHL also decreased expression of ICOS and BCL-6, two key regulators of Tfh cells (Supplementary Fig. 4d, e)^44^. Dual loss of *Vhl* and *Hif1a*, by targeting *Vhl* in *Hif1a*-KO Cas9^+^SMARTA cells, prevented both reductions in T cell expansion and defective Tfh differentiation seen in VHL-targeted cells (Fig. 3c, Supplemental Fig. 4c), directly implicating increased HIF-1α in these defects. Similar effects of *Vhl* and *Hif1a* deficiency were also observed at day 3 p.i., suggesting that increased HIF-1α is detrimental early during Tfh cell differentiation (Fig. 3d, Supplementary Fig. 4f).

To evaluate VHL regulation of Tfh differentiation in a different setting, we targeted both *Vhl* and *Hif1a* by double transduction using MRIG and a second sgRNA vector (MRIA) expressing an Ametrine fluorescent marker in Cas9^+^ OT-II TCR-transgenic T cells, followed by adoptive transfer into WT mice and NP-ovalbumin/alum immunization. Evaluation of doubly-transduced (GFP^+^Ametrine^+^) cells post-immunization confirmed the strong negative effect of VHL loss on CXCR5^+^PD-1^+^ Tfh cell generation that was counteracted by co-targeting *Hif1a* (Fig. 3e). Although we have not ruled out additional effects on Th1 cells, these data indicate that HIF-1α has repressive effects on Tfh cells that are observed in diverse immunological settings.

### HIF-1α alters glycolytic gene expression

**Fig. 4:**
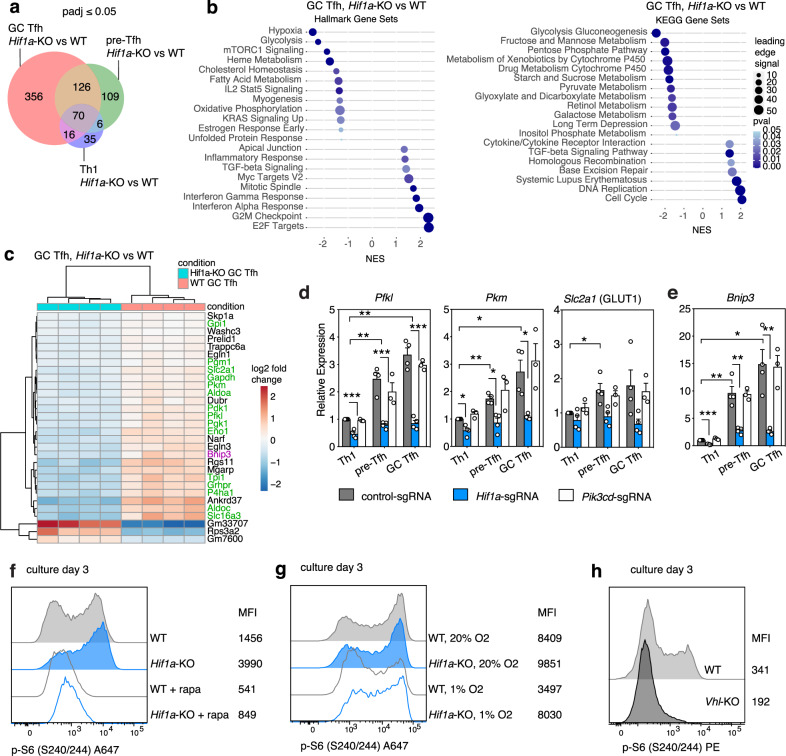
HIF-1α-mediated gene expression changes reveal negative regulation of mTORC1. **a** Venn diagram of differentially expressed genes from comparisons of *Hif1a*-KO and WT SMARTA Th1, pre-Tfh, and GC Tfh cell populations by bulk RNA-seq, on d8 post-LCMV. **b** GSEA analysis and **c** heatmap of differentially expressed genes from comparison of WT and *Hif1a*-KO SMARTA GC Tfh cells, on d8 post-LCMV. In (**c**), metabolic genes are highlighted in green, *Bnip3* is highlighted in purple. **d**, **e** qRT-PCR of select glycolytic genes (**d**) and *Bnip3* (**e**) mRNA in sorted p.i. SMARTA cells, as described in Fig. 2c. Data are pooled from *N* = 3–4 independent infection and sort experiments (control-sgRNA and *Hif1a*-sgRNA *N* = 4 experiments, *Pik3cd*-sgRNA *N* = 3). Data in (**d**, **e**) are presented as mean values +SEM. **f** Phospho-S6 (p-S6) staining in WT or *Hif1a*-KO SMARTA cells cultured under Tfh-like conditions, with or without rapamycin, on d3. **g** Phospho-S6 staining in WT or *Hif1a*-KO SMARTA cells cultured under Tfh-like conditions, under 20% or 1% O_2_, on d3. **h** Phospho-S6 staining in WT or *Vhl*-KO SMARTA cells cultured under Tfh-like conditions, on d3. Representative data for (**f**–**h**) shown from 1 of at least 2 independent experiments. Experimental details for (**d**, **e**) as described for Fig, 2c. *p < 0.05; **p < 0.01, ***p < 0.001 as evaluated by two-tailed unpaired Student’s *t* test (d-e). Source data provided in Source Data file and Supplementary Data 6.

To provide insight into mechanisms by which HIF-1α affects Tfh cells, we evaluated our RNA-seq data for genes that were differentially expressed between WT and *Hif1a*-KO Th1, pre-Tfh, and GC Tfh cells. The largest differential gene expression was seen between GC Tfh cell populations (Fig. 4a, Supplementary Table 6), consistent with the increased *Hif1a* transcripts in Tfh compared to Th1 cells. Along with hypoxia, the glycolytic transcriptional signature was one of the most significantly reduced in the *Hif1a*-KO versus WT SMARTA GC Tfh cells (Fig. 4b)—the top differentially expressed genes included many encoding proteins involved in glycolysis and related metabolic pathways (Fig. 4c, Supplementary Fig. 5a, b). qRT-PCR confirmed that several HIF-1α target genes associated with glycolysis (*Hk2*, *Pfkl*, *Gapdh*, *Pkm*, and *Slc2a1*, encoding the glucose transporter GLUT1), were higher in pre-Tfh and GC Tfh cells than Th1 cells and reduced in *Hif1a*-sgRNA cells (Fig. 4d, Supplementary Fig. 5c). Similarly, GLUT1 protein was reduced in *Hif1a*-sgRNA cells (Supplementary Fig. 5d), whereas GLUT1 protein was aberrantly high in Th1 cells lacking *Vhl*, and was partially normalized by the lack of both *Vhl* and *Hif1a* (Supplementary Fig. 5e); *Hif1a*-*Vhl* double-KO Tfh cells had reduced GLUT1 expression. Expression of *Hif1a*-target glycolytic genes was normal in *Pik3cd*-sgRNA SMARTA cells (Fig. 4d, Supplementary Fig. 5c, d), again suggesting that HIF-1α and PI3K pathways could be uncoupled in Tfh cells, similar to CD8 cytotoxic T lymphocytes^30^.

To evaluate whether glycolysis itself affected T helper cell differentiation in response to LCMV, we altered glucose transport by ectopic expression of GLUT1. Overexpression of GLUT1 did not normalize Tfh differentiation in *Hif1a*-KO SMARTA cells, but rather increased percentages of Tfh in both WT and *Hif1a*-KO cells relative to the vector control (Supplementary Fig. 6a), consistent with a previous report^3^. Furthermore, CRISPR targeting of GLUT1 failed to rescue decreased Tfh generation in *Vhl*-sgRNA cells (Supplementary Fig. 6b). Although we have not ruled out other effects of glycolytic enzymes^44^, or that a complete GLUT1-KO was too detrimental, these results suggested that HIF-1α may mediate its effect on Tfh development via additional or other downstream pathways.

### HIF-1α negatively regulates mTORC1

To understand other pathways affected by the HIF-1α/VHL axis, we further mined our RNA-seq analysis of *Hif1a*-KO versus WT GC Tfh cells. One of the most significant differentially expressed genes was *Bnip3* (Fig. 4c), a previously described transcriptional target of HIF-1α in T cells^45^; qRT-PCR confirmed *Bnip3* transcripts were markedly reduced in *Hif1a*-sgRNA cells (Fig. 4e). Moreover, like *Hif1a*, *Bnip3* transcripts were highest in GC Tfh and lowest in Th1 cells. Conversely, *Vhl*-sgRNA SMARTA cells exhibited higher BNIP3 protein post-LCMV infection (Supplementary Fig. 6c).

BNIP3 is a BCL-2 family member that is implicated in repressing mTORC1 in response to hypoxia^46^. While mTORC1 induces *Hif1a* in response to TCR signaling^30^, data also implicate HIF-1α in negative regulation of mTORC1^39^. To assess mTORC1 activation, we cultured naïve WT or *Hif1a*-KO SMARTA CD4^+^ T cells under Tfh-like inducing conditions, and measured phosphorylation of PI3K-AKT-mTOR pathway components. Staining for both p-AKT^S473^ and its downstream target p-FOXO1^S256^ were similar between WT and *Hif1a*-KO cells (Supplementary Fig. 6d), suggesting that *Hif1a*-KO cells have relatively intact PI3K activity, as well as activation of mTORC2, which phosphorylates AKT on S473. However, in *Hif1a*-KO cells, we observed increased p-S6^S240/244^, a downstream readout of mTORC1 (Fig. 4f), as confirmed by sensitivity to the mTORC1 inhibitor, rapamycin.

In contrast, WT cells cultured under 1% O_2_, which increases HIF-1α protein, showed reduced p-S6^S240/244^ compared to WT cells cultured at 20% O_2_ (Fig. 4g). *Hif1a*-KO cells failed to repress p-S6^S240/244^ in low O_2_, directly implicating HIF-1α in this negative regulation. Moreover, T cells cultured from *Vhl*^fl/fl^;Cd4-Cre (*Vhl*-KO) SMARTA T cells showed marked reductions in p-S6^S240/244^ compared to WT (Fig. 4h), further implicating the VHL- HIF-1α axis in the regulation of mTORC1 in T cells.

### HIF-1α promotes autophagic flux

mTORC1 drives anabolic metabolism in response to nutrient availability and mitogens; this is counterbalanced by macro-autophagy, which is activated during adverse microenvironmental conditions, including starvation and hypoxia^47^. Conversely, autophagy is suppressed by mTORC1. To evaluate autophagy, we transduced SMARTA cells with a fluorescent LC3b reporter, for which a higher GFP/RFP ratio indicates lower autophagic flux^48^. In Th1, pre-Tfh, and GC Tfh cells, loss of *Hif1a* decreased autophagic flux compared to controls (Supplementary Fig. 6e), consistent with a role of HIF-1α in repressing mTORC1 and promoting autophagy^49^.

### An expanded screen reveals multiple genes affecting Tfh cells

**Fig. 5:**
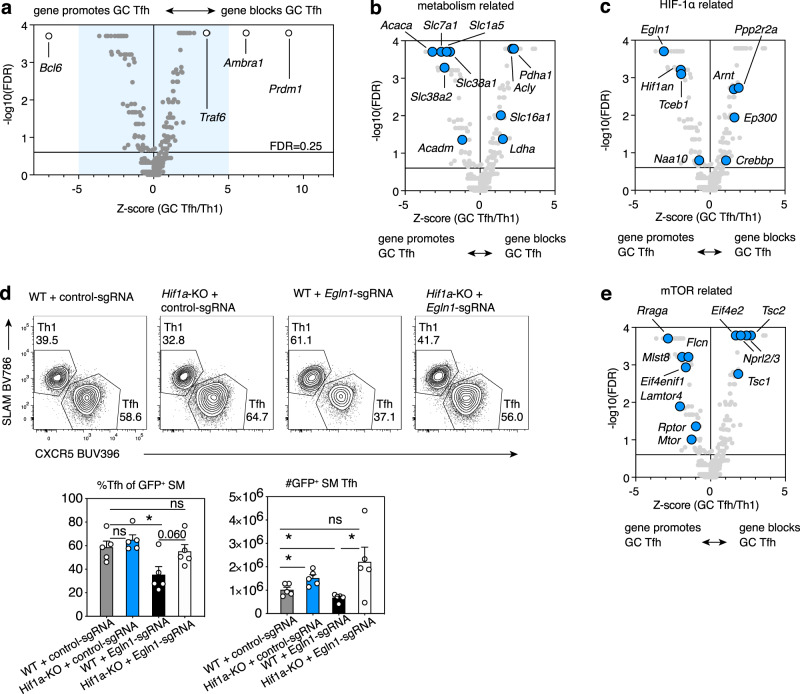
Opposing HIF-1α and mTORC1 signaling components affect Tfh differentiation. **a**–**c**, **e** An sgRNA library targeting a curated library of genes related to mTOR, HIF-1α, and autophagy signaling (Supplementary Data 7, 8) was screened from two separate cultures and sorts for d6 and 7 post-LCMV infection, *n* = 15 mice/day. Screen results were analyzed by Mageck for FDR and for L2FC to generate Z-scores. Shown are comparisons of GC Tfh to Th1 cells. Gene hits related to metabolism (**b**), HIF-1α (**c**), and mTOR (**e**) are highlighted in volcano plots comparing GC Tfh and Th1 cells. Blue area in (**a**) represents inset shown in (**b**, **c**, **e**). Each symbol represents the mean of all sgRNAs for one gene. **d** Representative flow plots, percentages, and numbers of GFP^+^ Tfh cells from WT or *Hif1a*-KO Cas9+ SMARTA cells transduced with the indicated sgRNAs, d6 post-LCMV infection., *n* = 5 mice/group. Data are presented as mean values +SEM. Representative data for (**d**) shown from 1 of 2 independent experiments. *p < 0.05 as evaluated by two-tailed unpaired Student’s *t* test (d). Source data provided in Source Data file and in Supplementary Data 8.

To expand our understanding of mTOR, HIF-1α, and related pathways in Tfh differentiation, we curated a library of ~2400 sgRNAs targeting ~600 genes related to these signaling networks to screen for effects in response to LCMV (Fig. 5a, Supplementary Fig. 7a, Supplementary Data 7). This screen confirmed the importance of these pathways, while serving as a resource of multiple genes that regulated Tfh versus Th1 cells. Among the top hits that increased the ratio of pre-Tfh and GC Tfh to Th1 cells were sgRNAs targeting genes encoding AMBRA1 and TRAF6, two proteins that together interact with the BECLIN1 complex to promote autophagy initiation^50^ (Fig. 5a and Supplementary Fig. 7a). However, both proteins are involved in multiple pathways and recent data show that AMBRA1 is an E3-Ubiquitin ligase involved in the degradation of Cyclin D and Myc^51–53^. SMARTA cells transduced with *Ambra1*-sgRNAs generated more Tfh cells in vivo than those transduced with control-sgRNA (Supplementary Fig. 7b).

Other significant hits provided evidence for differential metabolic and nutrient transport requirements of Tfh versus Th1 cells (Fig. 5b, Supplementary Fig. 7c). *Acaca* (which encodes a rate-limiting enzyme for fatty acid synthesis) strongly promoted pre-Tfh and GC Tfh differentiation versus Th1 (i.e. sgRNAs targeting this gene were depleted in Tfh cells, Fig. 5b, Supplementary Fig. 7c). Similarly, the cationic amino acid transporter CAT1 (*Slc7a1*), the neutral amino acid transporter ASCT2 (*Slc1a5*), and the glutamine transporters SNAT1/2 (*Slc38a1/2*), which are induced by Myc and required for mTOR activation^29,54^, all promoted Tfh cells. However, not all nutrient transporters and metabolic regulators were required for Tfh cell generation. SgRNAs directed against *Slc16a1*, a HIF-1α target encoding the MCT1 transporter for monocarboxylate metabolites (e.g., lactate, pyruvate), and LDHA, which interconverts lactate and pyruvate, were relatively increased in Tfh cells and depleted in Th1 cells (i.e., these genes inhibited Tfh cells relative to Th1 cells) (Fig. 5b, Supplementary Fig. 7c). Some hits highlighted potential metabolic differences between pre-Tfh and GC Tfh, e.g., *Slc38a2* strongly promoted GC Tfh relative to Th1, although this gene was a weaker hit for pre-Tfh versus Th1 cells; PDHA and ACLY, both involved in acetyl-CoA synthesis, inhibited GC Tfh relative to Th1 differentiation, but had less effect on pre-Tfh cells (Fig. 5b, Supplementary Fig. 7c). Together, these results suggest a strong metabolic component regulating Tfh versus Th1 differentiation.

### Opposing roles for HIF-1α and mTORC1 in Tfh cells

Among the significant hits affecting Tfh cells were multiple post-transcriptional regulators of HIF-1α, confirming the importance of this axis in Tfh cells. SgRNAs against *Egln1* (encoding PHD2, which hydroxylates HIF-1α for recognition by VHL), *Hif1an* (encoding FIH, which inhibits HIF-1α transcriptional activity), *Tceb1* (encoding Elongin C, part of the VHL complex), and *Naa10* (encoding ARD1a, which promotes HIF-1α association with VHL) were all depleted in pre-Tfh and GC Tfh relative to Th1 cells (Fig. 5c, Supplementary Fig. 7d). The products of these genes are involved in destabilization or inhibition of HIF-1α protein^38,55,56^, supporting our hypothesis that dampening HIF-1α is critical to allow maximal Tfh cell differentiation. In particular, PHD2 and FIH require O_2_ to destabilize/inhibit HIF-1α^38^, suggesting that their effects on Tfh differentiation may be influenced by environmental oxygen concentrations. Reduction of Tfh cells with *Egln1*-sgRNA was not observed in a HIF-1α-deficient background (Fig. 5d, Supplementary Fig. 7e), again implicating HIF-1α and hypoxia sensing in repression of Tfh cells.

In contrast, sgRNAs against *Ppp2r2a* (encoding the B55α regulatory subunit of Protein Phosphatase Type 2a, which stabilizes HIF-1α^57^), and *Arnt*, (encoding HIF-1β, the obligate partner of HIF-1α) were enriched in Tfh cells (Fig. 5c, Supplementary Fig. 7d). To mediate its transcriptional activity, HIF-1α/HIF-1β heterodimers associate with co-activators p300 and CBP^38,55^. SgRNAs against *Ep300*, and *Crebbp* (encoding p300, and CBP, respectively) were also increased in Tfh cells (Fig. 5c, Supplementary Fig. 7d); thus, these genes all restricted Tfh cell generation relative to Th1 cells.

At the same time, this screen emphasized the key roles of mTOR signaling in promoting Tfh cells (Fig. 5e and Supplementary Fig. 7f). SgRNAs targeting multiple genes involved in mTORC1/2 activation were depleted in (i.e., these genes were required for) Tfh cells, including *Rraga* (encoding the RagA GTPase, involved in lysosomal recruitment of mTORC1), *Mlst8* (a component of both mTORC1 and mTORC2 complexes) and *Lamtor4* (aka *C7orf59*, encoding part of the Ragulator complex) (Fig. 5e).

In contrast, sgRNAs targeting genes encoding multiple negative mTOR regulators, including Tuberous Sclerosis Complex (TSC)1 and 2, which sequester the mTORC1 activator RHEB, and NPRL2/NPRL3, parts of the GATOR1 complex that inactivates RagA/B^58^, were enriched in Tfh cells (i.e., loss of these genes increased Tfh cell generation, Fig. 5e and Supplementary Fig. 7f). We also observed higher levels of TSC2 protein in LZ versus DZ GC B cells, as well as in Tfh versus Th1 SMARTA cells, raising the possibility that modulation of its expression may help tune mTORC1 activity in GC B and Tfh cells (Supplementary Fig. 7g). Higher TSC2 protein was also seen in *Vhl*-sgRNA versus control SMARTA cells post-LCMV infection (Supplementary Fig. 7h), suggesting additional crosstalk between these signaling pathways.

### mTORC1 activation rescues Tfh:Th1 ratios in the absence of VHL

**Fig. 6:**
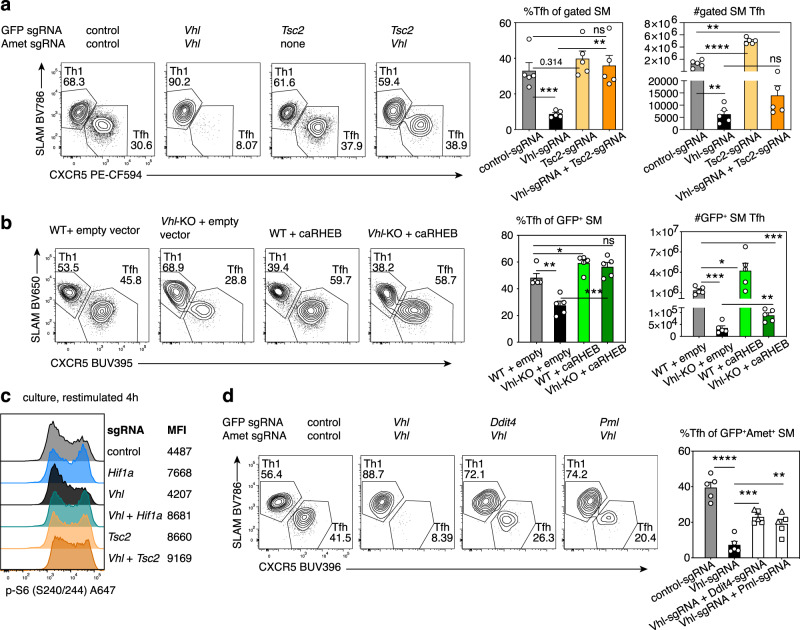
Increased mTORC1 activity rescues Tfh differentiation in *Vhl*-deficient T cells. **a** Representative flow plots, percentages of Tfh cells and total gated Tfh cells from Cas9^+^ SMARTA cells transduced with the indicated sgRNA vector(s), on d6 post-LCMV, *n* = 5 mice/group. SMARTA cells were gated on GFP^+^Ametrine^+^, except for the *Tsc2*-sgRNA group, which was gated on GFP^+^. **b** Representative flow plots, percentages of Tfh cells and total GFP^+^ Tfh cells from WT or *Vhl*-KO SMARTA cells transduced with empty or caRHEB-expressing vector, on d6 post-LCMV infection, *n* = 5 mice/group. **c** Phospho-S6 staining in Cas9^+^ SMARTA cells transduced with the indicated sgRNA vector(s) and restimulated in vitro with anti-CD3 plus anti-CD28 for 4 h on d5 postactivation. **d** Representative flow plots, percentages of Tfh cells from Cas9^+^ SMARTA cells transduced with the indicated sgRNA vector(s), d6 post-LCMV, *n* = 5 mice/group. For *Ddit4* and *Pml* sgRNAs, squares and triangles represent sgRNAs #1 and 2, respectively. Data in (**a**), (**b**), and (**d**) are presented as mean values + SEM. Representative data for (**a**-**d**) shown from 1 of at least 2 independent experiments. *p < 0.05; **p < 0.01, ***p < 0.001, ****p < 0.0001 as evaluated by two-tailed unpaired Student’s *t* test (**a**, **b**, **d**). Source data are provided in Source Data file.

If HIF-1α-mediated repression of Tfh cell differentiation resulted from mTORC1 dampening, we hypothesized that enhancing mTORC1 signaling should reverse defects in *Vhl*-deficient cells. Indeed, simultaneous deletion of the mTORC1 negative regulator *Tsc2* improved Tfh percentages in *Vhl*-sgRNA SMARTA cells (Fig. 6a). However, targeting *Tsc2* failed to fully rescue cell numbers (Fig. 6a, Supplementary Fig. 8a), suggesting that effects of *Vhl*-deficiency on Tfh differentiation can be at least partially uncoupled from effects on cell expansion. Similar results were seen by over-expressing constitutively active RHEB (caRHEB) in *Vhl*-KO (*Vhl*^fl/fl^;*Cd4*-Cre) SMARTA cells prior to transfer and LCMV infection (Fig. 6b); caRHEB rescued Tfh percentages although it did not rescue cell expansion (Fig. 6b, Supplementary Fig. 8b). Transduction with caRHEB also increased expression of ICOS in the absence of VHL (Supplementary Fig. 8c). Furthermore, while cultured *Vhl*-sgRNA T cells had attenuated p-S6^S240/244^ compared to control-sgRNA cells after in vitro restimulation with anti-CD3 and anti-CD28, S6 phosphorylation was increased by double transduction with either sgRNAs against *Hif1a* or *Tsc2* (Fig. 6c) or by caRHEB expression (Supplementary Fig. 8d). Thus, the balance of Tfh and Th1 cells appears to be regulated by HIF-1α/VHL-mediated tuning of mTORC1.

To investigate the mechanism behind mTORC1 inhibition by HIF-1α, we examined the effects of mutating *Bnip3* in *Vhl*-sgRNA cells. SgRNA-targeting of *Bnip3* was not sufficient to rescue Tfh generation (Supplementary Fig. 8e), nor did ectopic expression of BNIP3 decrease Tfh cell percentages in the context of HIF-1α deficiency (Supplementary Fig. 8f). However, HIF-1α represses mTORC1 via multiple intermediates, including 1) REDD1 (encoded by *Ddit4*), which activates TSC proteins, and 2) PML, which sequesters RHEB^47^. *Ddit4* was also a hit that inhibited Tfh cells in our druggable target screen (Fig. 2a). Targeting *Ddit4* or *Pml* partially restored percentages of Tfh cells in the absence of Vhl (Fig. 6d), suggesting they are critical intermediates downstream of HIF-1α However, they also failed to rescue cell expansion (Supplementary Fig. 8g).

### VHL/HIF-1α affect T cell expansion

**Fig. 7:**
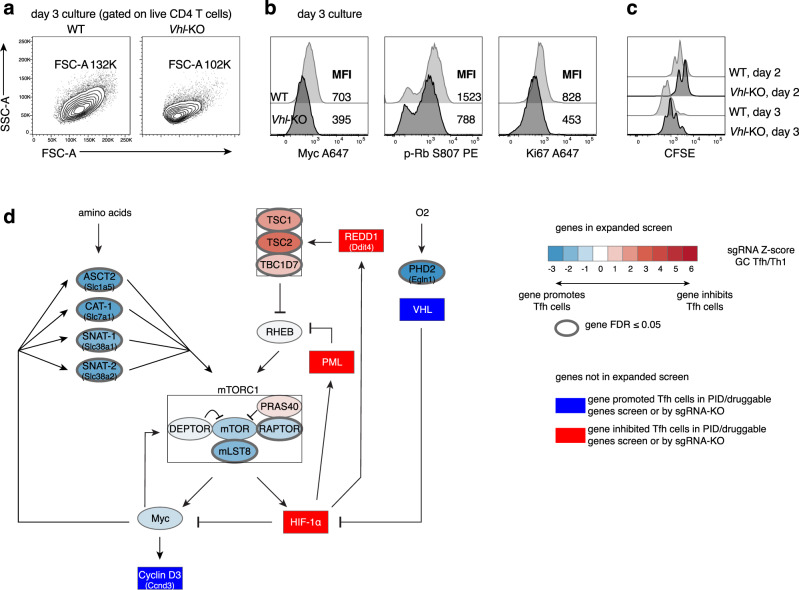
Vhl is required for optimal Myc expression and cell proliferation. **a**–**c** WT or *Vhl*-KO SMARTA cells were stained with CFSE, cultured under Tfh-like conditions, and stained and analyzed on d2 or 3 as indicated. **d** Schematic of HIF-1α-centered negative feedback loops with mTORC1 and Myc, which are also part of a positive feedback loop with each other. Blue: genes promoting Tfh cells; Red: genes inhibiting Tfh cells. Scale represented in legend in the figure. Representative data for (a–c) shown from 1 of at least 2 independent experiments.

The lack of full rescue of cell numbers by targeting TSC2 or expression of caRHEB in *Vhl*-KO cells suggested that HIF-1α overexpression has broader consequences beyond effects on mTORC1. One known target that is repressed by elevated HIF-1α is Myc, a key regulator of both metabolism and cell-cycle^59^. Upon T cell activation, Myc induces the expression of transporters for amino acids and other nutrients, which are required for T cell growth^60^. In Tfh-skewed cultures, *Vhl*-KO cells were smaller (Fig. 7a, Supplementary Fig. 9a), and had reduced Myc protein compared to WT (Fig. 7b), despite expressing more GLUT1 (Supplementary Fig. 9b). Myc is essential for T cell proliferation through regulation of cell cycle components, including induction of Cyclin D3, which phosphorylates and inactivates Rb (retinoblastoma tumor suppressor) to allow E2F family members to drive cell cycle progression^60^. *Vhl*-KO cells showed reduced p-Rb, Ki67, and delayed proliferation in culture (Fig. 7b, c). We also observed decreased proliferation and increased cell death of *Vhl*-sgRNA cells in response to LCMV, although both Tfh and Th1 cells were affected at d3 p.i. (Supplementary Fig. 9c-d). Conversely, cultured *Hif1a*-KO cells had higher Myc, p-Rb, and Ki67 (Supplementary Fig. 9e).

*Myc* transcripts were increased in both pre-Tfh and GC Tfh compared to Th1 populations post-LCMV infection (Supplementary Fig. 9f). Although *Myc* mRNA was not affected by the loss of HIF-1α in Tfh cells, two of the most enriched gene sets in *Hif1a*-KO versus WT GC Tfh cells were “Myc Targets V2” and “E2F Targets” (Fig. 4b), consistent with an inhibitory effect of HIF-1α on Myc protein and transcriptional activity. Furthermore, caRHEB did not rescue Myc levels or proliferation of *Vhl*-KO cells in culture (Supplementary Fig. 9g). Thus, loss of *Vhl* impairs both mTORC1 activation and cell proliferation via potentially distinct HIF-1α-dependent effects; although Myc levels are dependent on mTORC1^60^, increasing mTORC1 activity was not sufficient to rescue Myc expression and cell proliferation in the context of *Vhl*-deficiency. Finally, *Vhl*-KO cells also showed reduced p-AKT^S473^ and p-FOXO1^S256^ (Supplementary Fig. 9h), two downstream readouts of PI3K and mTORC2, while these markers were less affected in *Hif1a*-KO cells (Supplementary Fig. 6d). FOXO1 is a repressor of BCL-6 and Tfh cells that is inactivated by AKT-mediated phosphorylation downstream of ICOS^61^, and likely also contributes to impaired Tfh cell generation in *Vhl*-deficient cells, Thus, loss of VHL was more detrimental than the loss of HIF-1α.

Together, our results suggest that VHL is a critical member of a broad signaling network that regulates HIF-1α protein, which in turn tunes mTORC1 as well as optimal SMARTA CD4 cell expansion via both negative and positive feedback circuits (Fig. 7d and Supplementary Fig. 10). Thus, a carefully titrated amount of HIF-1α may be required to set the magnitude of the Tfh and germinal center response.

## Discussion

Through the use of targeted CRISPR-sgRNA libraries, cellular analyses, and epistatic evaluation of multiple gene sets, we have started to construct a picture of regulatory networks affecting Tfh cells, and have uncovered a requirement for the VHL/HIF-1α axis in setting the balance of Tfh cells versus Th1 cells during viral infection. The requirement for VHL in both acute viral infection and protein immunization models^44^ suggests that fine-tuning via HIF-1α-associated circuits may be a common feature of Tfh differentiation in multiple settings.

We linked our findings, in part, to inhibitory effects of HIF-1α on mTORC1; loss of VHL led to profound defects in p-S6 that could be reversed by simultaneous mutation of HIF-1α or by increasing mTORC1 activation. Our expanded screen further revealed multiple genes that oppose HIF-1α or promote mTOR activation were required for Tfh formation, whereas disrupting genes that activate HIF-1α or restraine mTOR increased Tfh:Th1 cell ratios.

Nonetheless, the effects of HIF-1α on Tfh cells are likely context-dependent. Two recent reports showed that HIF-1α expression in T cells was required for optimal Tfh and GC B cell generation after immunization;^35,36^ this was attributed in part to effects on Tfr cells^35^, effects we would not see using adoptively transferred SMARTA or OT-II TCR-transgenic cells. Yet, other data indicate that HIF-1α is dispensable for antibody titers after secondary boosting with NP-ovalbumin/alum^35^. Despite these differences, the profound effects of VHL-deficiency on Tfh generation provide evidence for a cell-intrinsic inhibitory effect of HIF-1α on Tfh cells. Our findings are supported by a study by Liu et al., which linked defects in Vhl-deficient cells to increased HIF-1α-mediated induction of GAPDH, and suppression of ICOS expression through m6A modification of *Icos* RNA^44^. We also observed reduced ICOS on *Vhl*-deficient cells, but found that increasing mTORC1 restored ICOS levels and did not observe differentiation defects in *Gapdh*-sgRNA cells in our expanded screen (Supplementary Data 8). While these differences may result from divergent models and the timing and extent of loss of these genes, complete KO of GAPDH sharply reduced in vivo SMARTA expansion, increasing noise in the Tfh/Th1 calculation in our screen and limiting its evaluation. Nonetheless, our studies both support critical roles for VHL/HIF-1α signaling in Tfh differentiation, and together, suggest the involvement of multiple mechanisms.

Using genetic knockout and hypomorphic models, mTORC1 and mTORC2 have been shown to promote Tfh differentiation by driving transcription of Tfh-essential transcription factors and a suite of metabolic genes^3,32–34^, as well as expression of and responses to ICOS^3^. However, there have also been divergent results on mTOR effects on Tfh cells in the literature, with two papers arguing these pathways are more important for driving Th1 responses^62,63^. MTORC1 is affected by multiple inputs, and has many downstream effectors other than HIF-1α^29^. Similarly, HIF-1α itself has activators in addition to mTORC1 and hypoxia, including reactive oxygen species, intermediate metabolites such as succinate^64^, and cytokines^38^, as well as multiple downstream effectors, which are also likely to affect Tfh cell differentiation. Nonetheless, the rescue of the balance of Tfh:Th1 cells by mTORC1 activation in VHL-deficient cells supports an epistatic relationship between the HIF-1α/VHL axis and mTORC1.

In addition to reductions in p-S6, *Vhl*-KO showed defects in cell growth and proliferation in culture, and in vivo post-LCMV infection-increasing mTORC1 activation was not sufficient to fully rescue these defects. Although mTORC1 affects cell growth and proliferation, HIF-1α can drive cell-cycle arrest through inhibition and degradation of Myc under conditions of hypoxia or *Vhl* deficiency^65^. How cell-cycle regulation affects Tfh cells is less clear, but Cyclin D3 was a top hit required for Tfh cells in our druggable target screen (Fig. 2a). Conversely, the most enriched gene set in *Hif1a*-KO GC Tfh cells compared to WT was targets of E2F, a family of cell-cycle regulatory effectors downstream of p-Rb that has been associated with Tfh and Tfh-like cells in both human^66,67^ and mice^68,69^. Both Myc and HIF-1α induce the expression of glycolytic machinery, but Myc also drives oxidative phosphorylation through mitochondrial biogenesis, whereas HIF-1α inhibits mitochondrial respiration^59^. Unregulated high levels of HIF-1α may therefore both lower Myc protein and uncouple glycolysis from oxidative phosphorylation. Tfh cells have higher mitochondrial mass than Th1 cells^19^, whereas *Vhl*-KO SMARTA cells have severely reduced mitochondrial mass^44^, supporting HIF-1α-driven alterations in mitochondrial metabolism in Tfh cells. Finally, decreased p-AKT and p-FOXO1 implicate decreased mTORC2 activity in Tfh defects in *Vhl*-deficient cells, as that FOXO1 restrains Tfh cells via repression of *Bcl6*. Decreased expression of ICOS, which induces activation of PI3K and its downstream effector, AKT^70^, likely contributes to these phenotypes. The wide range of defects in *Vhl*-KO cells suggests that HIF-1α protein must be tightly regulated to generate appropriate Tfh responses, likely by influencing multiple factors affecting Tfh differentiation and expansion.

It is therefore relevant that our expanded screen revealed multiple related factors required for Tfh cell differentiation, including numerous metabolic regulators. These included amino acid transporters Slc7a1, Slc38a1/2, and Slc1a5, which are induced by Myc^71^ and are required to activate mTORC1 and drive effector functions in T cells^72^. Also notable, AMBRA1 was a top hit increasing Tfh differentiation when targeted with sgRNAs. Although AMBRA1 is implicated in the early stages of autophagy^73^, AMBRA1 is also an E3-Ubiquitin ligase responsible for degrading Cyclin D^51–53^ and Myc^74^. Our screen therefore supports intimately connected roles for metabolic, nutrient sensing, cell-cycle, and related pathways in Tfh generation.

CRISPR/Cas9 has become a important tool for target discovery in T cells, including evaluation of T cell signaling, activation, and proliferation^75,76^. Although the requirement for cell expansion in response to infection limits the numbers of sgRNAs we could evaluate in vivo, our use of curated targeted libraries provides a powerful tool to interrogate specific pathways or classes of proteins. Our work complements other in vivo studies^77–83^ by taking advantage of a rapid screen for T cell differentiation in a physiological setting that does not require a selective advantage^77^. Furthermore, our highly efficient vector system allowed the targeting of multiple individual genes, facilitating genetic complementation and network analyses.

The regulatory circuitry of Tfh cells has remarkable overlap with those affecting GC B cells—including the requirement for BCL-6 and repression by BLIMP-1. Similarly, Cyclin D3 is required for driving GC B cell proliferation^28,84^. It is therefore of interest that several publications suggest that the GC is a hypoxic environment^39–41^, particularly in the light zone (LZ) where Tfh cells are enriched^85^. In support of these data, we find higher expression of multiple HIF-1α target genes in WT Tfh cells compared to Th1 cells; similar results were seen in recent single-cell RNA-seq studies of LCMV-specific CD4 T cells^24,86^. Nonetheless, GCs are likely not severely hypoxic, since constitutively high HIF-1α in *Vhl*-KO cells is detrimental for Tfh development, leading to impaired activation of mTORC1 and decreased Myc, which are also key regulators of GC B cells^37,42^. MTORC1 activity along with Myc increase in LZ GC B cells in response to Tfh cell help and drive progression to the DZ and proliferation of GC B cells^37^. Similarly, high-affinity interactions with B cells induce Myc, mTORC1, and proliferation in Tfh cells^87^. It is intriguing to speculate that HIF-1α may help provide a check on mTORC1 and Myc activity in both GC B and Tfh cells until productive positive-selecting interactions occur. Such negative feedback circuitry may help restrain Tfh numbers and promote stringency of B cell selection; this may be particularly important for Tfh cells, which require repeated antigenic stimulation for their generation^88^ and proliferation^87^. High numbers of Tfh cells can be associated with autoimmunity and paradoxical decreases in antigen-specific GC B cell responses^26,89^; restraining Tfh cell numbers, through the activities of PD-1, HIF-1α, and other inhibitory molecules, may be a key quality control. Furthermore, the reliance of these circuits on oxygen and nutrient availability may allow Tfh cells to integrate microenvironmental cues with antigenic signals. Whether specific nodes of these networks can be perturbed for therapeutic purposes remains an intriguing question.

## Methods

### Mice

C57BL6/J, *Hif1a*-flox^90^ (007561), *Vhl*-flox^91^ (012933), *Cd4*-Cre^92^ (022071), constitutive Cas9^13^ (024858), CD45.1 congenic (002014), and *Bcl6*-flox^93^ mice (023727) on a C57Bl6/J background were from Jackson Labs. SMARTA TCR LCMV-specific mice^16^ on a C57Bl6 background, (gift of Dr. Ethan Shevach, NIAID), were crossed to Cas9 transgenic and CD45.1 mice to obtain Cas9 SMARTA CD45.1 mice. Appropriate strains were bred to obtain *Hif1a*-flox*Cd4*-Cre Cas9 SMARTA CD45.1 mice, *Vhl*-flox*Cd4*-Cre SMARTA CD45.1 mice and *Hif1a*-flox *Vhl*-flox *Cd4*-Cre SMARTA CD45.1 mice. Animal husbandry and experiments were performed under specific-pathogen-free conditions in accordance with protocols approved by Animal Use and Care Committees of the National Human Genome Research Institute (NHGRI protocol G98.3) or National Institutes of Neurological Diseases and Stroke (NINDS protocol 1295-21), National Institutes of Health, Animal Welfare Assurance #A-4149-01. Euthanasia was performed by CO2 inhalation followed by cervical dislocation. Either male or female mice between 2 and 4 months age were utilized for experiments. Controls were age- and sex-matched and whenever possible co-housed littermates were used. Within each experiment, all mice were bred and housed in the same facility and room.

### Reagents

Primer sequences for cloning and fluorescent PCR, CRISPR sgRNA sequences, and real-time PCR primers, are listed in Supplementary Data 1. Detailed lists of the flow cytometry reagents, including antibodies, are listed in the Reporting Summary and Supplementary Data 9.

### Plasmids

To construct the MRCIG vector, the mU6-sgRNA fragment (BglII-SalI), and the SV40-spCas9 fragment (SalI – EcoRI) of pQCiG2^11^ were subcloned into MIGR1 (Addgene 27490), upstream of IRES-GFP. To clone MRIG, the BglII-SalI mU6-sgRNA fragment from pQCiG2 was inserted into MIGR1 after the latter was digested with BglII and XhoI upstream of IRES-GFP.

MRIA was generated by PCR amplifying the Ametrine ORF from pAmetrine-N1 (Addgene 54505), and subcloning into MRIG using NcoI and SalI sites.

The GFP-LC3-RFP-LC3ΔG fragment of pMRX-IP-GFP-LC3-RFP-LC3ΔG^48^ (Addgene 84572) was subcloned into MSCV IRES Thy1.1 using BglII and NotI sites.

The pMIG GLUT1-myc IRES GFP overexpression vector was a gift of Dr. Jeff Rathmell (Vanderbilt). The constitutively active RHEB overexpression construct was cloned by inserting a 5′-FLAG-tagged mouse RHEB CDS (Addgene #13831) into MIGR1 using NEBuilder HiFi DNA assembly kit (NEB). The N153T mutation was introduced by QuikChange kit (Agilent Technologies). The BNIP3 overexpression construct was cloned by inserting the CDS from Myc-BNIP3FL (Addgene 100796) into MIGR1 using restriction site cloning.

### Retroviral transduction, adoptive transfer, infection, immunization

293T cells (ATCC) were cultured in EMEM (ATCC) + 10% fetal bovine serum + 2 mM L-glutamine (Thermo) and transfected using TransIT-293 (Mirus) according to manufacturer instructions. Viral supernatants were collected at 24 and 48 h and spun at 2500 rpm for 10 min at 4 C to remove cellular debris.

Naïve SMARTA or OT-II were isolated using the naïve CD4 T cell kit (Miltenyi), cultured in RPMI with 10% FCS, and transduced with retroviral vectors as described^12,15^. Briefly, naïve T cells were activated on anti-CD3 and anti-CD28 (8 ug/ml each for SMARTA, 5 ug/ml each for OT-II) coated plates. Viral supernatant containing 10 U/ml hIL-2 and 8 ug/ml polybrene (Sigma) were added at 24 and 40 h postactivation, and spun at 37 °C for 90 min at 2500 rpm. Media was then replaced with RPMI 10% FCS plus 10 U/ml hIL-2. Double transductions were performed with a 1:1 ratio of the two viral supernatants.

OT-II cells were transferred on day 3 postactivation. SMARTA cells were cultured with 10 U/ml hIL-2 for 2 additional days, then on day 5 postactivation, replated with 2 ng/ml IL-7 (Peprotech) overnight. Just prior to transfer, the percentage of live GFP^+^ or GFP^+^Ametrine^+^ cells was measured by flow cytometry. For transduced SMARTA cells, 1 × 10^6^ or 1 × 10^5^ fluorescent cells were transferred for analysis on day 3 and days 6–8 p.i., respectively. For transduced OT-II cells, 4 × 10^5^ fluorescent cells were transferred. For naïve SMARTA transfers, 1 × 10^6^ or 1 × 10^4^ cells were transferred into WT hosts for analysis on day 3 and day 8 p.i., respectively. 2 × 10^4^ cells were transferred into *Bcl6*^fl/fl^*Cd4*-Cre hosts for analysis on day 8 p.i. On the indicated day p.i., spleens were harvested, and single-cell suspensions were stained Th1 and Tfh cells. Transferred transduced cells were gated for GFP^+^ or GFP^+^Ametrine^+^ cells.

OT-II recipients were immunized i.p. with 50 ug NP_16_-ovalbumin (Biosearch) in Imject Alum (Thermo) 2 days post-transfer. Recipients that received naïve SMARTA cells were infected 1 day post-transfer. Recipients receiving activated SMARTA cells were infected 3–4 days post-transfer. SMARTA recipients were infected with 2 × 10^6^ or 2 × 10^5^ pfu of LCMV Armstrong i.v., for analysis on day 3 or days 6–8, respectively. Tfh and Th1 cells were evaluated in the GFP^+^ or GFP^+^Ametrine^+^ cells.

For overexpression of proteins, naïve SMARTA cells were transduced as described above at 24 h postactivation, rested for 1 h in the incubator, and 1 × 10^5^ cells were immediately transferred into WT hosts, which were infected with LCMV 2 days later.

### Fluorescent PCR fragment analysis

Live cells were sorted according to GFP (Supplementary Fig. 11) and target protein expression, and genomic DNA was extracted by the DNEasy Blood and Tissue kit (Qiagen). DNA was PCR amplified around the target cut sites using a three-primer reaction using two gene-specific primers and a common fluorescent primer (Supplementary Data 1) to generate approximately 300 bp fluorescent-labeled amplicons, and reaction products were run on a capillary sequencer (Genetic Analyzer 3130xl)^14^.

### Single and pooled library sgRNA construct cloning

SgRNA sequences were either manually designed using Benchling or used from the Brie library^94^. Sequences were ordered as individual oligos (IDT) containing a partial mU6 sequence and partial tracrRNA sequence at the 5′ and 3′ ends respectively^12^: 5′- ggagaaaagccttgtttg-N20-gttttagagctaggatcctagc (N20 indicates the spacer sequence). For libraries, 5 oligos/gene (PID library, Supplmentary Data 2) or 4 oligos/gene (druggable target, Supplementary Data 4 and expanded screen libraries, Supplementary Data 7) were either ordered individually as above (IDT) and manually combined at equimolar ratios, or ordered as a pool (Twist Bio) with extended 5′ and 3′ sequences: caattggagaaaagccttgtttg-N20-gttttagagctaggatcctagcaagtt. Single or pooled oligos were PCR amplified using ARRAY-F and ARRAY-R primers (Supplementary Data 1) and purified using Qiaquick PCR kits^12^. The MRIG backbone was digested with BamHI-HF, MfeI-HF, treated with shrimp alkaline phosphatase, and gel purified^12^, then ligated with sgRNA oligo amplicons by HiFi assembly at a 1:5 backbone:insert molar ratio. For single sgRNA constructs, HiFi reactions were diluted 1:5 in water and 1 ul was transformed into 20 ul HB101 competent cells (Zymo). For libraries, HiFi reactions were diluted 1:5 in water, and 1 ul was electroporated into 20 ul Stbl4 (Thermo), in quadruplicate, following manufacturer instructions. Bacteria were shaken in SOC for 1.5 h at 37 C to recover, then plated on LB agar-ampicillin bioassay plates (Nunc) for 24 h at 30 C. Colonies per sgRNA coverage were 143x for the PID genes library, 86x for the druggable targets library, and 230x for the mTOR-HIF-1α expanded library. Colonies were scraped and plasmids purified using Qiagen EndoFree Maxiprep kits.

### CRISPR screens

Cas9^+^ SMARTA cells were cultured as described above for individual sgRNAs, except with only one round of transduction at 24 h postactivation.

The PID genes screen was performed as two independent experiments. In each experiment, 3–4 × 10^6^ cultured cells were saved prior to transfer for DNA isolation, and 10 WT recipients received 1 × 10^6^ GFP^+^ SMARTA cells each. Mice were euthanized on day 6 and day 7 postinfection, 5 mice per day. For each mouse, cells from the spleen and peripheral lymph nodes were pooled, and each mouse was sorted separately for CXCR5 versus SLAMF1 (referred to as SLAM). Four samples were selected from the first experiment and five samples from the second for deep sequencing. Coverage for each sgRNA was ≥750x sorted cells and ≥300x sequencing depth for all samples.

The druggable genes screen was performed once. 6 × 10^6^ cultured cells were saved prior to transfer. Seventeen WT recipients received 1 × 10^6^ GFP^+^ SMARTA cells each. Mice were euthanized on day 6 and day 7 postinfection, 8–9 mice per day. Spleens and peripheral lymph nodes were harvested separately, then pooled from 4–5 mice for sorting for CXCR5 versus PD-1. Coverage for each sgRNA was ≥1000x sorted cells and ≥1200x sequencing depth for all samples.

The expanded mTOR-HIF-1α-related screen was performed with two parallel cultures for day 6 and day 7 p.i. For each culture, 5 × 10^5^ cultured cells were saved prior to transfer and 15 WT recipients received 8 × 10^5^ GFP^+^ SMARTA cells each. Mice were euthanized on day 6 or day 7 postinfection, 15 mice per day. Spleens were pooled from all mice each day for sorting for CXCR5 versus PD-1. Coverage for each sgRNA was ≥3000x sorted cells and ≥1000x sequencing depth for all samples.

Sorted cell pellets were resuspended in 100 ul PBS and stored at −80C. Genomic DNA was purified from sorted cells using Qiagen DNEasy kits. Genomic DNA was used for PCR amplification of ~600 bp surrounding the sgRNA sequence using guide ID primers (Supplementary Data 1). A second round of PCR added indexing primers to samples for multiplex next-generation sequencing (Supplementary Data 1). Samples were purified by E-gel (Thermo), quantified by Qubit (Thermo), then sequenced on an Illumina NextSeq 500 using a NextSeq 500/550 High Output v2 kit (75 cycles) (Illumina #FC-404-2005) following manufacturer instructions. Counts of sgRNAs were extracted from FASTQ files and normalized using a custom perl script^95^ and Bowtie2 with the following parameters: -p 16 -f–local -k 10–very-sensitive-local -L 9 -N 1. Custom scripts are available at https://lymphochip.nih.gov/local/CRISPR/ or upon request. For the PID genes screen, L2FC values were calculated manually for each replicate, then averaged across replicates per experiment, then averaged across experiments. For the druggable genes screen and expanded screen, L2FC values and false-discovery rates were calculated by Mageck v0.5.9^96^ using the control sgRNA and paired comparison options, then Z-scores were calculated manually by averaging L2FC values of all sgRNAs.

### In vitro cell culture

To differentiate in vitro Tfh-like cells, naïve CD4 T cells were isolated from spleen and lymph nodes of WT or Hif1a-KO mice, and plated in IMDM 10% FCS with 10 ug/ml each of anti-IFN-g, anti-IL-4, anti-IL-12, anti-TGF-b (all from Bioxcell), 100 ng/ml IL-6, and 50 ng/ml IL-21 (both cytokines from Peprotech), on plates coated with 3 ug/ml anti-CD3 and 5 ug/ml anti-CD28. For inhibitor studies, CAL-101 (Santa Cruz Bio) was added at 4 nM from the beginning of culture, rapamycin (Calbiochem) was added at 10 nM for the last 24 h of culture. For hypoxia studies, cultures were incubated in a hypoxic cabinet (Coy Labs) supplied with a 94/5/1 N_2_/CO_2_/O_2_ mixture.

### Flow cytometry

Spleen or peripheral lymph nodes were placed in nylon mesh cell strainers in FACS buffer (PBS + 0.5% BSA) and dissociated with a 3 ml syringe plunger to obtain single-cell suspensions. Spleen samples were resuspended in 1 ml ACK (Ammonium Chloride) lysis buffer and incubated for 2 min at room temperature, then quenched with 5 ml FACS buffer and filtered through 70-micron filter. Cells were washed once and resuspended in FACS buffer. CXCR5 staining was performed with unconjugated primary, biotin goat anti-rat secondary, and fluorescent-conjugated streptavidin in PBS + 0.5% BSA + 2% FCS + 2% normal mouse serum (Sigma). For intracellular protein staining, cells were fixed with 4% paraformaldehyde (PFA) and permeabilized with Permeabilization/Wash buffer (Thermo). For transcriptional factor staining, cells were first fixed with 2% PFA to preserve fluorescent proteins if needed, then fixed with Foxp3 Fixation/Permeabilization buffer (Thermo), and permeabilized with Permeabilization/Wash buffer.

For IL-21 and CD40L staining, splenocytes from LCMV-infected mice were plated in RPMI-10 with 1:1000 GolgiStop (BD), 1 ug/ml LCMV gp61 peptide (Anaspec), and fluorophore-conjugated anti-CD40L antibody (1:500). Unstimulated cells were plated similarly but without peptide. Cells were incubated at 37 C for 4 h, stained with viability dye and antibodies against surface markers, fixed in BD CytoFix/CytoPerm, permeabilized with Permeabilization/Wash buffer, and stained with IL-21R/Fc and anti-human Fc secondary antibody.

For hypoxyprobe staining, pimonidazole (60 mg/kg) or saline was injected into mice 1 h prior to euthanasia. After surface marker and viability dye staining, cells were fixed in 4% PFA and permeabilized in Permeabilization/Wash buffer, then stained with the Hypoxyprobe-PAb27 kit antibody for 1 h at 4 C, followed by antirabbit secondary for 30 min at 4 C. For each cell population of interest, averaged hypoxyprobe MFI from saline-injected mice was subtracted from that of the test mouse. For cell proliferation analysis, cells were stained with CFSE or CellTrace Violet (Thermo) at 1 uM for in vitro experiments or 5 uM for in vivo experiments according to manufacturer instructions.

Cells were analyzed on an LSR II or Fortessa (BD), data were collected in FACSDiva v8 and analyzed using FlowJo v10 (Treestar). Cells were sorted on a FACSAria (BD). Post-sort fractions had higher than 95% purify, as verified by flow cytometry analysis on the same FACSAria machine used to sort the cells.

### Phospho-staining

For Tfh-like cultures, on d2 or d3 postactivation (to allow expression of VHL and HIF1a which are not expressed in naïve cells), cells were stained with fixable viability dye, fixed in 4% paraformaldehyde, permeabilized in methanol at −20C for at least 1 h, washed with PBS once, then stained in FACS buffer with phospho-protein antibodies.

SgRNA or control transduced T cells were cultured in fresh complete RPMI 10% FCS with 10 U/ml hIL-2 daily, from day 3 to day 5 postactivation. Cells were rested in RPMI 1% FCS for 2 h, replated in fresh RPMI 1% FCS and restimulated by adding 1 ug/ml anti-CD3, 3 ug/ml anti-CD28, and 5 ug/ml goat anti-hamster (Jackson Immunoresearch) for 4 h. Cells were stained with viability dye, fixed, permeabilized, and stained as described above.

### qRT-PCR

Sorted cell pellets were frozen in Trizol (Thermo), and total RNA was purified using the Qiagen RNEasy kit. cDNA was made using Taqman Reverse Transcription reagents (Thermo). qRT-PCR reactions were set up using Taqman Universal PCR Master Mix or PowerUp SYBR Green Master Mix (Thermo) following manufacturer instructions. Reactions were performed on a QuantStudio 6 (Applied Biosystems). *Actb* (b-actin) was used for normalization. Primers for qRT-PCR are listed in Supplmentary Data 1.

### RNA-seq and differential gene expression analysis

Th1, pre-Tfh, and GC Tfh were sorted from naïve SMARTA transferred mice at day 8 p.i. Sorted cell pellets were frozen in Trizol, and total RNA was purified using the Qiagen RNEasy kit. RNA-seq libraries were prepared with 1 ug total RNA per sample using the TruSeq RNA Library Preparation Kit v2, Set A and Set B (Illumina #RS-122-2001 and RS-122-2002) following manufacturer instructions, and sequenced on an Illumina NextSeq 500 using a NextSeq 500 High Output v2 kit (150 cycles) (Illumina #FC-404-2005).

Raw paired-end FASTQ reads for each sample were quality trimmed using Trimmomatic tool, version 0.3^97^ using the following settings: PE ILLUMINCLIP:Truseq3-PE.fa:2:30:10 LEADING:10 TRAILING:10 SLIDINGWINDOW:4:15 MINLEN:36. Quality trimmed reads were aligned to Ensembl Mus musculus genome assembly GRCm38.94 using STAR alignment tool version 2.5.3^98^. STAR alignment was run including –quantMode TranscriptSam flag to generate read counts per gene using Ensembl GRCm38.94 annotation to define gene features. Sample gene counts were analyzed for differential expression using the Bioconductor 3.10 package DESeq2 1.26^99^. Pathway enrichment plots were generated using GSEA^100^ v4, using normalized counts data (normCounts) from DESeq2 as the input GCT file. RNAseq analyses by DESeq2 used the Wald test to generate p-values, which are adjusted for multiple testing using the Benjamini-Hochberg procedure. We used an adjusted p-value cutoff of 0.05 for significance (Supplemental Table 6). RNA-seq data is available in GEO (GSE144467).

### Statistics

Data were analyzed in Prism 8 (GraphPad). Unless otherwise stated, all representative data came from one of at least two independent experiments, and mean values are shown with s.e.m. error bars. Student’s two tailed *t*-test was used for pairwise comparisons. P-values are annotated as follows: ns not significant, *p < 0.05, **p < 0.01, ***p < 0.001, ****p < 0.0001. RNAseq and library analyses are described above.

### Reporting summary

Further information on research design is available in the Nature Research Reporting Summary linked to this article.

## Supplementary information

Supplementary Information

Description of additional Supplementary File

Supplementary Data 1

Supplementary Data 2

Supplementary Data 3

Supplementary Data 4

Supplementary Data 5

Supplementary Data 6

Supplementary Data 7

Supplementary Data 8

Supplementary Data 9

Reporting Summary

## Data Availability

All data are included in the Supplemental data and Source Data files, or available from the authors upon reasonable requests, as are unique reagents used in this Article. Source data are provided with this paper. All parental mouse strains, including SMARTA mice (030450) are commercially available from Jax. CRISPR constructs and libraries are available from the authors upon reasonable request. RNA-seq data is deposited at GEO under accession number GSE144467. Source data are provided with this paper.

## Source data

Source Data File

## Code availability

Custom scripts for analyses of CRISPR screens are available at [https://lymphochip.nih.gov/local/CRISPR/]^95^
