# Supplementary material for: In vivo CRISPR screens reveal a HIF-1α-mTOR-network regulates T follicular helper versus Th1 cells
Source: Nat Commun. 2022 Feb 10;13:805. doi: 10.1038/s41467-022-28378-6 (PMC8831505; doi:10.1038/s41467-022-28378-6)
Supplement: Supplementary file 1 — Supplementary Information [file 41467_2022_28378_MOESM1_ESM.pdf]

Supplementary Material for:

In vivo CRISPR screens reveal HIF-1α-mTOR-mediated regulation of T follicular helper versus Th1 cells

Bonnie Huang….Pamela L. Schwartzberg

* Corresponding Authors
Pamela L. Schwartzberg pams@nih.gov
Bonnie Huang bonnie.huang@nih.gov

FIG S1

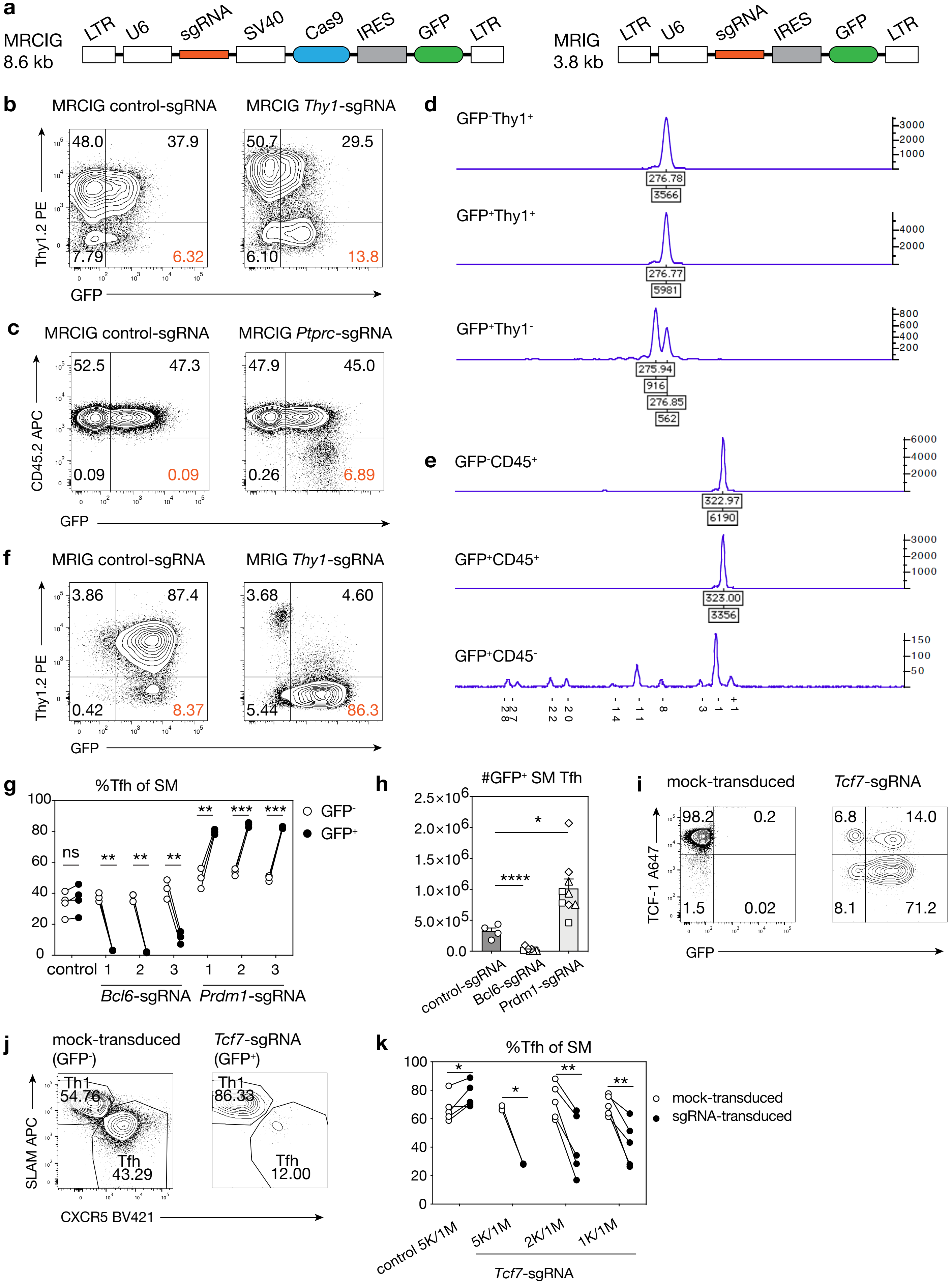
a
MRCIG 8.6 kb
LTR U6 sgRNA SV40 Cas9 IRES GFP LTR
MRIG 3.8 kb
LTR U6 sgRNA IRES GFP LTR
b
MRCIG control-sgRNA
MRCIG Thy1-sgRNA
Thy1.2 PE
GFP
48.0 37.9 7.79 6.32
50.7 29.5 6.10 13.8
c
MRCIG control-sgRNA
MRCIG Ptprc-sgRNA
CD45.2 APC
GFP
52.5 47.3 0.09 0.09
47.9 45.0 0.26 6.89
d
GFP-Thy1+
276.78
3566
GFP+Thy1+
276.77
5981
GFP+Thy1-
275.94
916
276.85
562
e
GFP-CD45+
322.97
6190
GFP+CD45+
323.00
3356
GFP+CD45-
-28 -27 -22 -20 -14 -11 -8 -3 -1 +1
f
MRIG control-sgRNA
MRIG Thy1-sgRNA
Thy1.2 PE
GFP
3.86 87.4 0.42 8.37
3.68 4.60 5.44 86.3
g
%Tfh of SM
GFP-
GFP+
ns ** ** ** ** *** ***
control 1 2 3 1 2 3
Bcl6-sgRNA Prdm1-sgRNA
h
#GFP+ SM Tfh
* ****
control-sgRNA
Bcl6-sgRNA
Prdm1-sgRNA
i
mock-transduced
Tcf7-sgRNA
TCF-1 A647
GFP
98.2 0.2 1.5 0.02
6.8 14.0 8.1 71.2
j
mock-transduced (GFP-)
Tcf7-sgRNA (GFP+)
SLAM APC
CXCR5 BV421
Th1 54.76
Tfh 43.29
Th1 86.33
Tfh 12.00
k
%Tfh of SM
* * ** **
control 5K/1M
5K/1M 2K/1M 1K/1M
Tcf7-sgRNA
mock-transduced
sgRNA-transduced

Supplementary Figure 1. Additional characterization of CRISPR-mediated gene knockout in primary mouse T cells

a, Schematic of MRCIG and MRIG vectors. **b**, Thy1.2 expression of WT CD4 T cells transduced with MRCIG control- or *Thy1*-sgRNA after 6 days in culture. **c**, Same experimental setup as (b), CD45.2 expression with control- or *Ptprc*-sgRNA. **d**, Fluorescent PCR *Thy1* amplicon size analysis of subpopulations of the cells from (b) sorted by GFP and Thy1.2 expression. **e**, Same experimental setup as (d), *Ptprc* amplicons from cells sorted from (c). **f**, Thy1.2 expression of $Cas9^+$ CD4 T cells transduced with MRIG control- or *Thy1*-sgRNA after 6 days in culture. **g**, Matched GFP^- and GFP^+ comparisons of Tfh percentages from $Cas9^+$ SMARTA cells transduced with control-, *Bcl6*-, or *Prdm1*-sgRNA, in each LCMV-infected WT host from the experiment in Fig. 1c. Control-sgRNA n=4 mice, all other sgRNAs n=3. **h**, Total number of GFP^+ Tfh cells in (g), replicates pooled per sgRNA target. For *Bcl6* and *Prdm1* sgRNAs, squares, triangles, and diamonds represent sgRNAs #1, 2, and 3, respectively, with similar results for all sgRNAs. Control-sgRNA n=4 mice, *Bcl6*- and *Prdm1*-sgRNA n=9 mice. Data presented as mean +SEM. **i**, TCF-1 expression of $Cas9^+$ SMARTA CD4 T cells transduced with MRIG control- or *Tcf7*-sgRNA after 6 days in culture. **j**, Representative flow plots of Tfh differentiation of cells from (i) after transfer into WT hosts, on day 6 post-LCMV infection. **k**, $Cas9^+$ SMARTA cells transduced with MRIG *Tcf7*-sgRNA were spiked into mock-transduced cells at the indicated frequencies, and the mixture was transferred into WT hosts for LCMV infection. Matched mock-transduced and GFP^+ comparisons of %Tfh among SMARTA cells from each host on day 6 post-LCMV infection. n=2 mice for 5K *Tcf7*-sgRNA group, n=5 for all other groups. Representative data shown from 1 of 2 independent experiments. *$p<0.05$; **$p<0.01$, ***$p<0.001$, ****$p<0.0001$ as determined by two-tailed paired (g, k) or unpaired (h) Student's *t* test. Source data are provided in Source Data file.

FIG S2

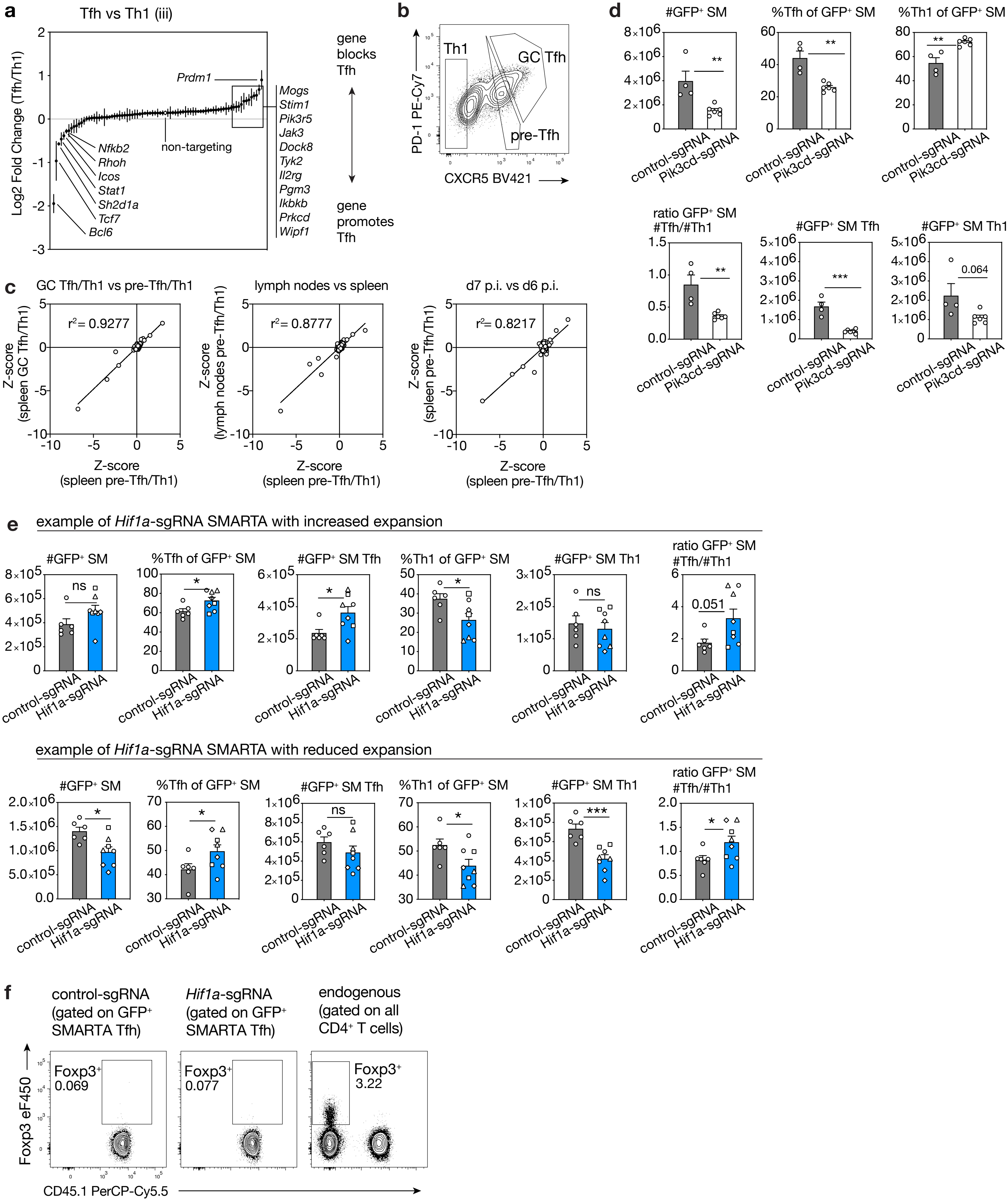

a
Tfh vs Th1 (iii)
Log2 Fold Change (Tfh/Th1)
Prdm1
Mogs
Stim1
Pik3r5
Jak3
Dock8
Tyk2
Il2rg
Pgm3
Ikbkb
Prkcd
Wipf1
Nfkb2
Rhoh
Icos
Stat1
Sh2d1a
Tcf7
Bcl6
non-targeting
gene blocks Tfh
gene promotes Tfh
b
PD-1 PE-Cy7
CXCR5 BV421
Th1
GC Tfh
pre-Tfh
c
GC Tfh/Th1 vs pre-Tfh/Th1
lymph nodes vs spleen
d7 p.i. vs d6 p.i.
r²= 0.9277
r²= 0.8777
r²= 0.8217
Z-score (spleen GC Tfh/Th1)
Z-score (lymph nodes pre-Tfh/Th1)
Z-score (spleen pre-Tfh/Th1)
d
#GFP⁺ SM
%Tfh of GFP⁺ SM
%Th1 of GFP⁺ SM
ratio GFP⁺ SM #Tfh/#Th1
#GFP⁺ SM Tfh
#GFP⁺ SM Th1
0.064
control-sgRNA
Pik3cd-sgRNA
e
example of Hif1a-sgRNA SMARTA with increased expansion
example of Hif1a-sgRNA SMARTA with reduced expansion
ns
0.051
control-sgRNA
Hif1a-sgRNA
f
control-sgRNA (gated on GFP⁺ SMARTA Tfh)
Hif1a-sgRNA (gated on GFP⁺ SMARTA Tfh)
endogenous (gated on all CD4⁺ T cells)
Foxp3⁺ 0.069
Foxp3⁺ 0.077
Foxp3⁺ 3.22
Foxp3 eF450
CD45.1 PerCP-Cy5.5

Supplementary Figure 2. PID and druggable genes screen details; additional characterization of *Pik3cd*- and *Hif1a*-sgRNA SMARTA cells *in vivo*

a, Waterfall plot from Fig. 1h with additional genes labeled (all genes ≥1.2-fold relative enrichment or depletion in Tfh versus Th1). **b**, Gates for sorting Th1, pre-Tfh, and GC Tfh cells in SMARTA cells on day 6-8 p.i. **c**, Correlations between sgRNAs Z-scores from the druggable target genes screen as performed and analyzed in Fig. 2a. Each symbol represents one gene, averaged over all its sgRNAs. R^2 values were calculated using Prism least squares regression fitting, without slope or y-intercept constraints. **d**, Quantification of GFP^+ SMARTA, Tfh, and Th1 cells from control- versus *Pik3cd*-sgRNA transduced $Cas9^+$ SMARTA cells, on day 6 p.i. post-LCMV infection, n=4 mice for control-sgRNA, 6 for *Pik3cd*-sgRNA. **e**, Total GFP^+ SMARTA numbers, Tfh percentages and cell numbers, Th1 percentages and cell numbers, and ratios of Tfh:Th1 cells from two independent experiments of control- versus *Hif1a*-sgRNA transduced $Cas9^+$ SMARTA cells, on day 6 post-LCMV infection, n=6 mice for control-sgRNA, 8 for *Hif1a*-sgRNA. For *Hif1a* sgRNAs, circles, squares, triangles, and diamonds represent sgRNAs #1, 2, 3, and 4, respectively. **f**, Representative flow plots of Foxp3 staining in control- or *Hif1a*-sgRNA SMARTA cells, after transfer into WT hosts, compared to host endogenous GFP^{neg} CD4 T cells, on day 7 post-LCMV infection. Data in (d) and (e) are presented as mean values +SEM. Representative data for (d-f) shown from 1 of at least 2 independent experiments. *p<0.05; **p<0.01, ***p<0.001, as evaluated by two-tailed unpaired Student's *t* test (d-e). Source data are provided in Source Data file and Supplementary Data 3 and 5.

FIG S3

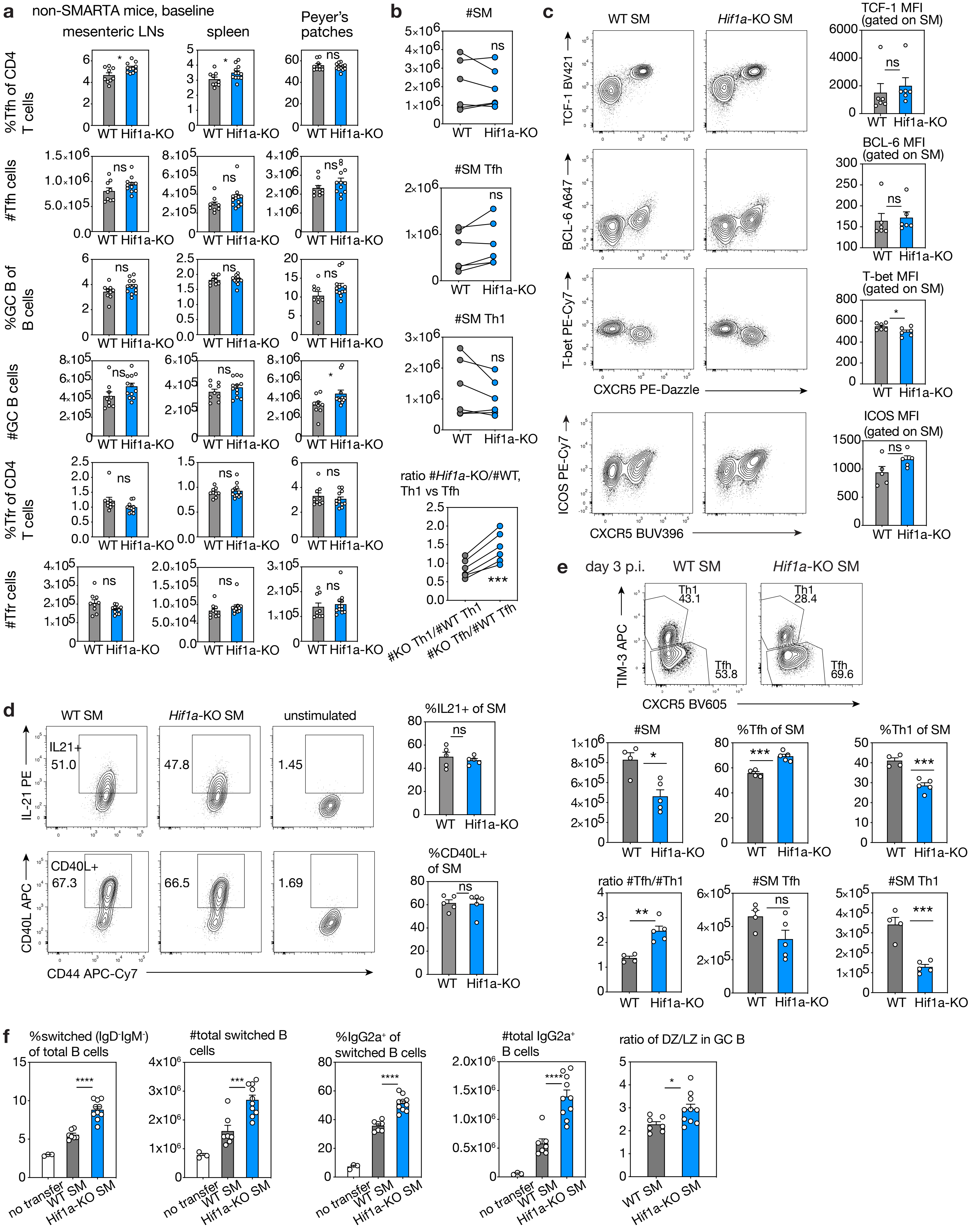

a
non-SMARTA mice, baseline
mesenteric LNs
spleen
Peyer's patches
%Tfh of CD4 T cells
#Tfh cells
%GC B of B cells
#GC B cells
%Tfr of CD4 T cells
#Tfr cells
WT
Hif1a-KO
b
#SM
#SM Tfh
#SM Th1
ratio #Hif1a-KO/#WT, Th1 vs Tfh
#KO Th1/#WT Th1
#KO Tfh/#WT Tfh
c
WT SM
Hif1a-KO SM
TCF-1 BV421
BCL-6 A647
T-bet PE-Cy7
CXCR5 PE-Dazzle
ICOS PE-Cy7
CXCR5 BUV396
TCF-1 MFI (gated on SM)
BCL-6 MFI (gated on SM)
T-bet MFI (gated on SM)
ICOS MFI (gated on SM)
d
WT SM
Hif1a-KO SM
unstimulated
IL21+
51.0
47.8
1.45
IL-21 PE
CD40L+
67.3
66.5
1.69
CD40L APC
CD44 APC-Cy7
%IL21+ of SM
%CD40L+ of SM
e
day 3 p.i.
WT SM
Hif1a-KO SM
Th1 43.1
Tfh 53.8
Th1 28.4
Tfh 69.6
TIM-3 APC
CXCR5 BV605
#SM
%Tfh of SM
%Th1 of SM
ratio #Tfh/#Th1
#SM Tfh
#SM Th1
f
%switched (IgD-IgM-) of total B cells
#total switched B cells
%IgG2a+ of switched B cells
#total IgG2a+ B cells
ratio of DZ/LZ in GC B
no transfer
WT SM
Hif1a-KO SM

Supplementary Figure 3. Additional characterization of and Hif1a-KO polyclonal and SMARTA cells *in vivo*

a, Baseline percentages and numbers of Tfh, GC B, and Tfr cells in mesenteric lymph nodes, spleen, and Peyer's patches of WT or $Hif1a^{fl/fl}$*Cd4*-Cre mice, pooled from 2 independent experiments, n=9 mice for WT, 11 for *Hif1a*-KO except n=10 for mesenteric LN Tfh and Tfr plots. Tfh cells were gated as $CD4^{+}B220^{-}CXCR5^{+}PD\text{-}1^{+}Foxp3^{-}$. GC B cells were gated as $B220^{+}CD19^{+}Fas^{+}GL\text{-}7^{+}$, Tfr cells were gated as $CD4^{+}B220^{-}CXCR5^{+}PD\text{-}1^{+}Foxp3^{+}$. **b**, Quantification of the experiment in Fig. 2d: total SMARTA, Tfh, and Th1 numbers, and the ratio of KO to WT for Tfh and for Th1 populations within each recipient. **c**, Representative flow plots of TCF-1, BCL-6, T-bet and ICOS staining, in WT versus *Hif1a*-KO SMARTA cells, on day 8 post-LCMV infection, and quantification of MFI in total SMARTA population, n=6 mice/group for TCF-1, BCL-6 and T-bet stains, n=5 mice/group for ICOS stains. **d**, Representative flows plots of intracellular IL-21 and surface CD40L staining after *in vitro* restimulation of WT versus *Hif1a*-KO SMARTA cells, on day 7 post-LCMV infection, and quantification of percentages in total SMARTA population, n=5 mice/group. **e**, Representative flow plots, Tfh and Th1 percentages and total numbers, and ratio of total Tfh to Th1 cells, in WT versus *Hif1a*-KO SMARTA, on day 3 post-LCMV infection, n=4 mice for WT, 5 for *Hif1a*-KO. **f**, Frequencies and total numbers of isotype switched ($IgD^{-}IgM^{-}$) B cells and $IgG2a^{+}$ B cells, ratio of dark zone to light zone in GC B cells, in the experiment in Fig. 2f. Data in (a), (c)-(f) are presented as mean values +SEM. Representative data for (b-f) shown from 1 of at least 2 independent experiments. *p<0.05; **p<0.01, ***p<0.001, ****p<0.0001 as evaluated by two-tailed unpaired Student's *t* test. Source data are provided as a Source Data file.

FIG S4

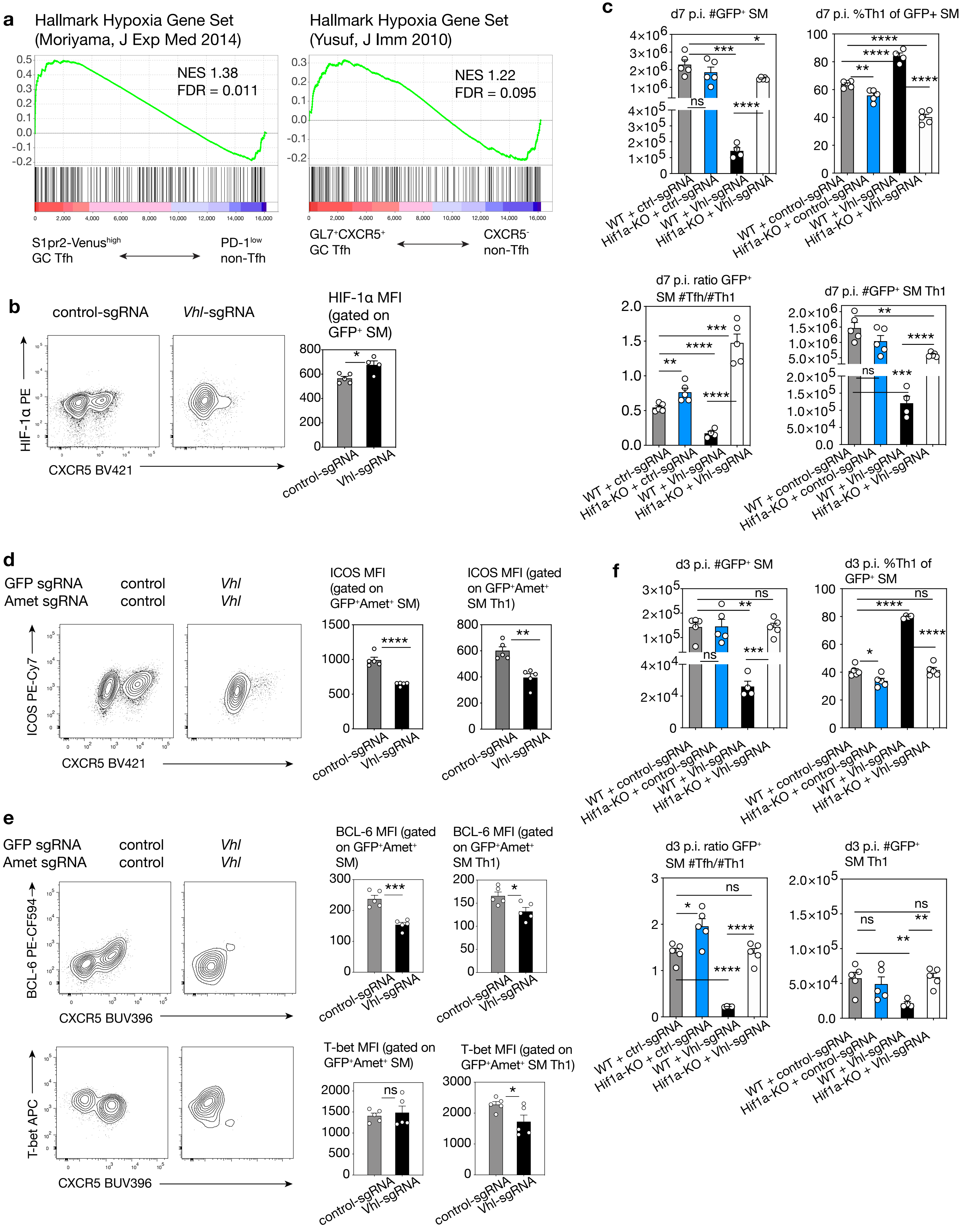
a
Hallmark Hypoxia Gene Set (Moriyama, J Exp Med 2014)
NES 1.38
FDR = 0.011
S1pr2-Venushigh GC Tfh
PD-1low non-Tfh
Hallmark Hypoxia Gene Set (Yusuf, J Imm 2010)
NES 1.22
FDR = 0.095
GL7+CXCR5+ GC Tfh
CXCR5- non-Tfh
b
control-sgRNA
Vhl-sgRNA
HIF-1α PE
CXCR5 BV421
HIF-1α MFI (gated on GFP+ SM)
c
d7 p.i. #GFP+ SM
d7 p.i. %Th1 of GFP+ SM
d7 p.i. ratio GFP+ SM #Tfh/#Th1
d7 p.i. #GFP+ SM Th1
WT + ctrl-sgRNA
Hif1a-KO + ctrl-sgRNA
WT + Vhl-sgRNA
Hif1a-KO + Vhl-sgRNA
WT + control-sgRNA
Hif1a-KO + control-sgRNA
d
GFP sgRNA control Vhl
Amet sgRNA control Vhl
ICOS PE-Cy7
CXCR5 BV421
ICOS MFI (gated on GFP+Amet+ SM)
ICOS MFI (gated on GFP+Amet+ SM Th1)
e
BCL-6 PE-CF594
CXCR5 BUV396
BCL-6 MFI (gated on GFP+Amet+ SM)
BCL-6 MFI (gated on GFP+Amet+ SM Th1)
T-bet APC
T-bet MFI (gated on GFP+Amet+ SM)
T-bet MFI (gated on GFP+Amet+ SM Th1)
f
d3 p.i. #GFP+ SM
d3 p.i. %Th1 of GFP+ SM
d3 p.i. ratio GFP+ SM #Tfh/#Th1
d3 p.i. #GFP+ SM Th1

Supplementary Figure 4. Characterization of *Vhl*-sgRNA SMARTA cells *in vivo*

a, GSEA analysis of the "Hallmark Hypoxia" gene set using publicly available Tfh gene expression data (Moriyama GSE56883 and Yusuf GSE21380). **b**, Representative flow plots of HIF-1a staining in Cas9$^+$ SMARTA cells transduced with control- or *Vhl*-sgRNA, transferred into WT hosts, on day 6 post-LCMV infection. n=5 mice for control-sgRNA, n=4 for *Vhl*-sgRNA. **c**, total number of GFP$^+$ SMARTA cells, percent GFP$^+$ SMARTA Th1 cells, total GFP$^+$ SMARTA Th1 cells, and ratio of #GFP$^+$ SM Tfh to #GFP$^+$ SM Th1 cells, from the experiment in Fig. 3c. **d**, Representative flow plots of ICOS and **e**, BCL-6 and T-bet staining and MFI in Cas9$^+$ SMARTA cells transduced with control- or *Vhl*-sgRNAs, on day 6 post-LCMV infection. For MFIs, populations were gated on total GFP$^+$Amet$^+$ SMARTA cells or on GFP$^+$Amet$^+$ SMARTA Th1 cells (CXCR5$^-$SLAM$^+$). n=5 mice/group. **f**, total number of GFP$^+$ SMARTA cells, percent GFP$^+$ SMARTA Th1 cells, total GFP$^+$ SMARTA Th1 cells, and ratio of #GFP$^+$ SM Tfh to #GFP$^+$ SM Th1 cells, from the experiment in Fig. 3d. Data in (b-f) are presented as mean values +SEM. Representative data for (b-f) shown from 1 of at least 2 independent experiments. *p<0.05; **p<0.01, ***p<0.001, ****p<0.0001 as evaluated by two-tailed unpaired (b-f) Student's *t* test. Source data are provided as a Source Data file.

FIG S5

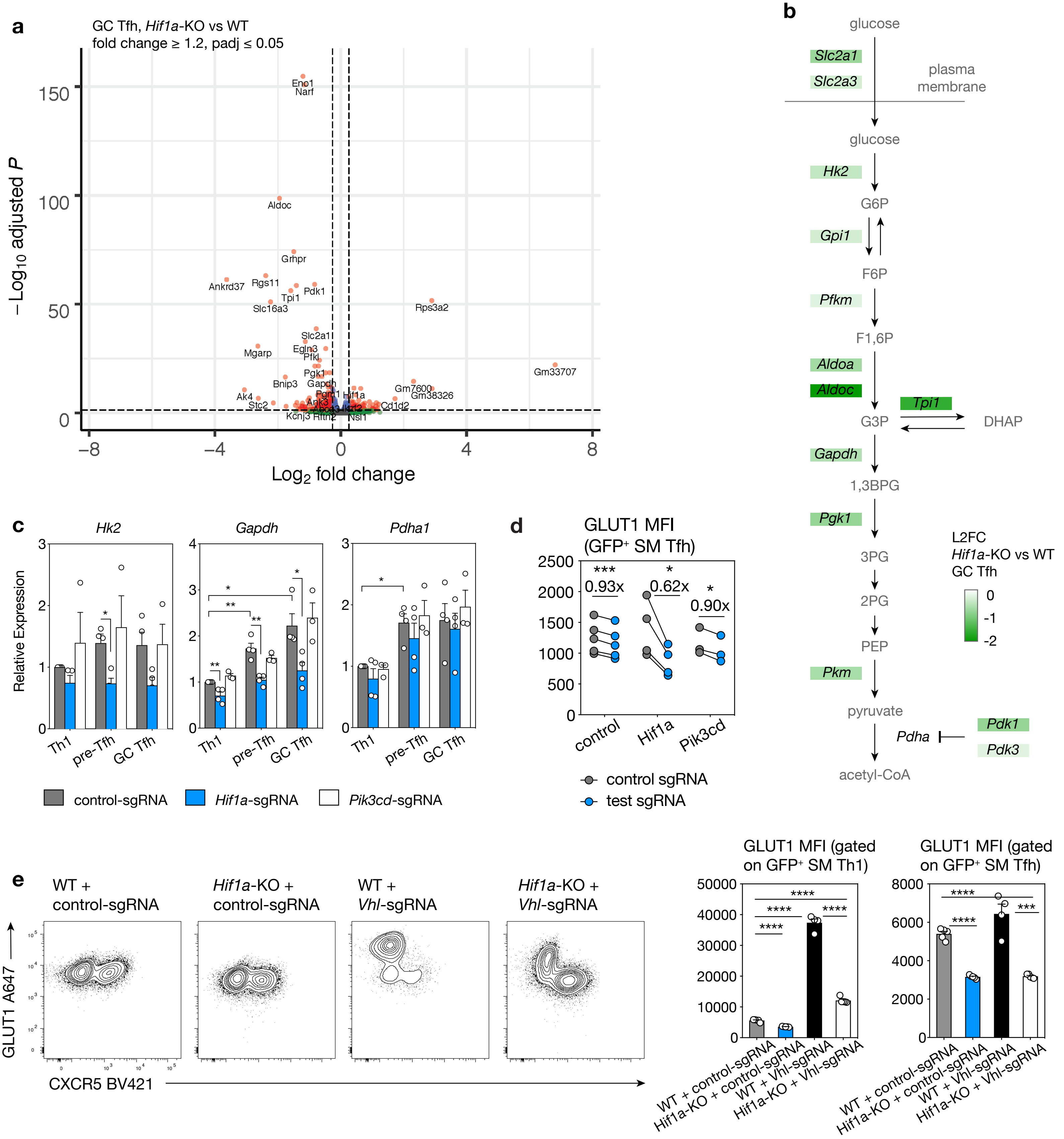
a
GC Tfh, Hif1a-KO vs WT
fold change ≥ 1.2, padj ≤ 0.05
−Log10 adjusted P
Log2 fold change
Eno1
Narf
Aldoc
Grhpr
Ankrd37
Rgs11
Pdk1
Tpi1
Slc16a3
Rps3a2
Slc2a1
Egln3
Pfkl
Mgarp
Pgk1
Bnip3
Gapdh
Gm33707
Gm7600
Gm38326
Pgm1
Hif1a
Ak4
Stc2
Ank3
Cd1d2
Kcnj3
Nsl1
b
glucose
Slc2a1
Slc2a3
plasma membrane
glucose
Hk2
G6P
Gpi1
F6P
Pfkm
F1,6P
Aldoa
Aldoc
Tpi1
G3P
DHAP
Gapdh
1,3BPG
Pgk1
3PG
2PG
PEP
Pkm
pyruvate
Pdha
Pdk1
Pdk3
acetyl-CoA
L2FC
Hif1a-KO vs WT
GC Tfh
c
Hk2
Gapdh
Pdha1
Relative Expression
Th1
pre-Tfh
GC Tfh
control-sgRNA
Hif1a-sgRNA
Pik3cd-sgRNA
d
GLUT1 MFI
(GFP+ SM Tfh)
0.93x
0.62x
0.90x
control
Hif1a
Pik3cd
control sgRNA
test sgRNA
e
WT + control-sgRNA
Hif1a-KO + control-sgRNA
WT + Vhl-sgRNA
Hif1a-KO + Vhl-sgRNA
GLUT1 A647
CXCR5 BV421
GLUT1 MFI (gated on GFP+ SM Th1)
GLUT1 MFI (gated on GFP+ SM Tfh)
WT + control-sgRNA
Hif1a-KO + control-sgRNA
WT + Vhl-sgRNA
Hif1a-KO + Vhl-sgRNA

Supplementary Figure 5. HIF-1a related gene expression changes *in vivo*

a, Volcano plot of differentially expressed genes and **b**, schematic of select glycolysis pathway components, from comparison of WT and *Hif1a*-KO GC Tfh SMARTA cells by RNA-seq, on day 8 post-LCMV infection. In (b), metabolites are in grey, genes are color-coded in green by RNA-seq expression difference. **c**, qRT-PCR measurement of select glycolytic gene mRNA levels in sorted post-LCMV infection $Cas9^+$ SMARTA cells, as in Fig. 3c. **d**, Comparison of total GLUT1 protein staining MFI in Tfh cells, comparing co-transferred CD45.2/.2 $Cas9^+$ SMARTA cells transduced with control-sgRNA and CD45.1/.2 $Cas9^+$ SMARTA cells transduced with the indicated test sgRNA vectors, on day 6 post-LCMV infection. n=5 mice for control-sgRNA, 4 for *Hif1a*-sgRNA, 3 for *Pik3cd*-sgRNA. **e**, Representative flow plots of total GLUT1 protein staining and MFI in Th1 and Tfh cells, in WT or *Hif1a*-KO $Cas9^+$ SMARTA cells transduced with control- or *Vhl*-sgRNA vector, on day 6 post-LCMV infection, n=4 mice for *Vhl*-sgRNA, 5 for all other groups. Data in (c) and (e) are presented as mean values +SEM. RNA-seq analyses in (a) used the Wald test to generate p-values, which were adjusted for multiple testing using the Benjamini-Hochberg procedure. Representative data for (d-e) shown from 1 of at least 2 independent experiments. *p<0.05; **p<0.01, ***p<0.001, ****p<0.0001 as evaluated by two-tailed unpaired (c, e) or paired (d) Student's *t* test. Source data are provided as a Source Data file and in Supplementary Data 6.

FIG S6

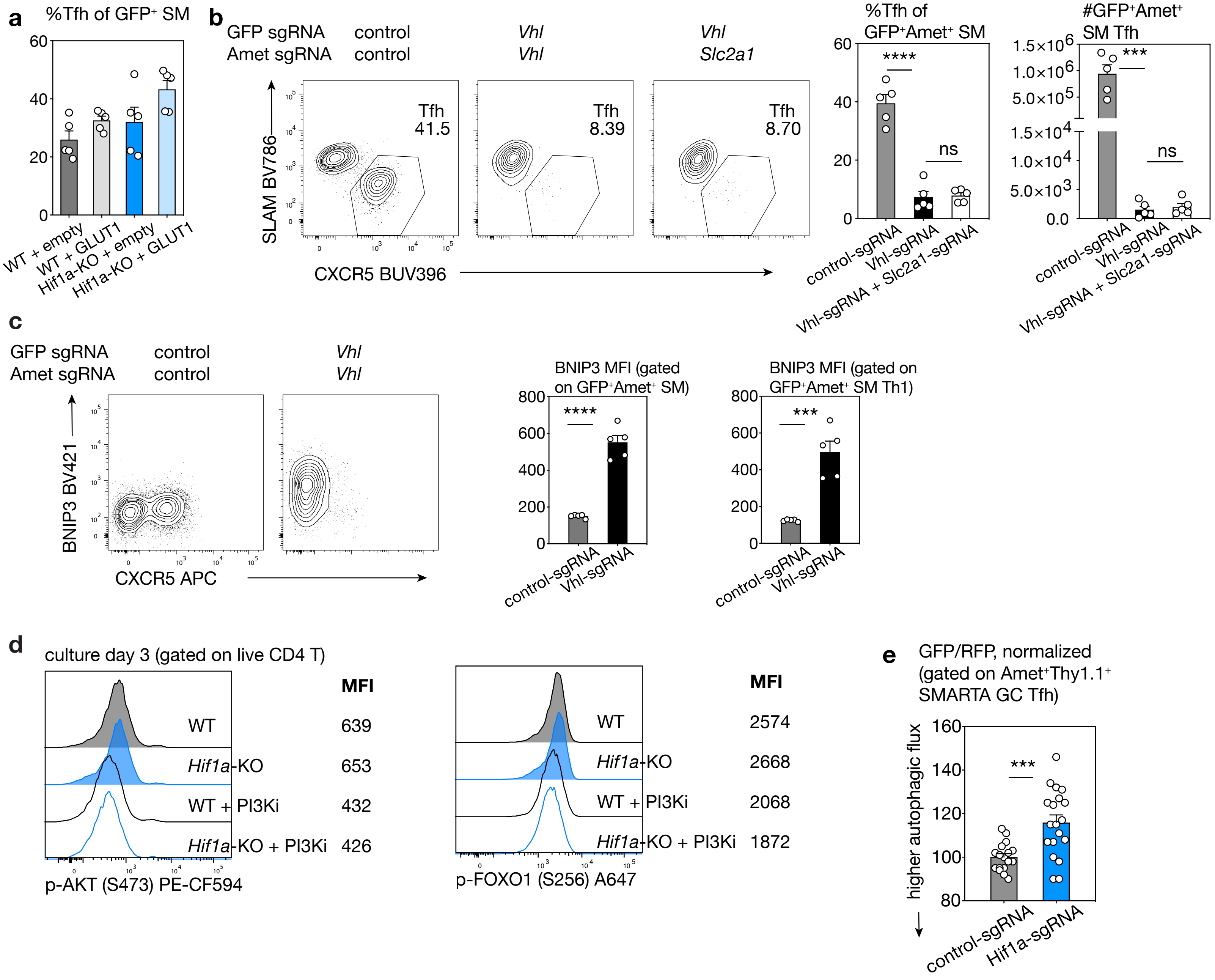

a
%Tfh of GFP+ SM
WT + empty
WT + GLUT1
Hif1a-KO + empty
Hif1a-KO + GLUT1
b
GFP sgRNA
Amet sgRNA
control
control
Vhl
Vhl
Vhl
Slc2a1
Tfh
41.5
Tfh
8.39
Tfh
8.70
SLAM BV786
CXCR5 BUV396
%Tfh of GFP+Amet+ SM
****
ns
#GFP+Amet+ SM Tfh
***
ns
control-sgRNA
Vhl-sgRNA
Vhl-sgRNA + Slc2a1-sgRNA
c
GFP sgRNA
Amet sgRNA
control
control
Vhl
Vhl
BNIP3 BV421
CXCR5 APC
BNIP3 MFI (gated on GFP+Amet+ SM)
****
BNIP3 MFI (gated on GFP+Amet+ SM Th1)
***
control-sgRNA
Vhl-sgRNA
d
culture day 3 (gated on live CD4 T)
MFI
WT 639
Hif1a-KO 653
WT + PI3Ki 432
Hif1a-KO + PI3Ki 426
p-AKT (S473) PE-CF594
MFI
WT 2574
Hif1a-KO 2668
WT + PI3Ki 2068
Hif1a-KO + PI3Ki 1872
p-FOXO1 (S256) A647
e
GFP/RFP, normalized (gated on Amet+Thy1.1+ SMARTA GC Tfh)
higher autophagic flux
***
control-sgRNA
Hif1a-sgRNA

Supplementary Figure 6. HIF-1a downstream signaling pathways

a, Tfh percentages in GFP^{+} WT or *Hif1a*-KO SMARTA cells transduced with empty or GLUT1-expressing retroviral vectors, on day 6 post-LCMV infection. n=5 mice/group. **b**, Representative flow plots, Tfh percentages and total GFP^{+}Ametrine^{+} Tfh cells, in Cas9^{+} SMARTA cells transduced with indicated sgRNAs, on day 6 post-LCMV infection. The data in (**b**) came from the same experiment as the one in Fig. 6d. **c**, Representative flow plots of BNIP3 staining in Cas9^{+} SMARTA cells transduced with control- or *Vhl*-sgRNAs, on day 6 post-LCMV infection, n=5 mice/group. For MFIs, populations were gated on total GFP^{+}Amet^{+} SMARTA cells or on GFP^{+}Amet^{+} SMARTA Th1 cells (CXCR5^{-}SLAM^{+}). **d**, Phospho-AKT and phospho-FOXO1 staining in WT or *Hif1a*-KO SMARTA T cells cultured under Tfh-like conditions, with or without PI3Kd inhibitor CAL-101, at day 3. **e**, LC3b GFP/RFP ratios in GC Tfh cells within Cas9^{+} SMARTA cells co-transduced with a LC3b reporter and control- or *Hif1a*-sgRNA vector, on day 6 post-LCMV infection. Values are pooled from 3 independent experiments n=17 total control; n=19 total *Hif1a*-sgRNA. GFP/RFP ratios within each experiment were normalized to the average of control-sgRNA samples. Data in (a)-(c) and (e) are presented as mean values +SEM. Representative data for (a-e) shown from 1 of at least 2 independent experiments. ***p<0.001, ****p<0.0001 as evaluated by two-tailed unpaired Student's t test (b, c, e). Source data are provided as a Source Data file.

FIG S7

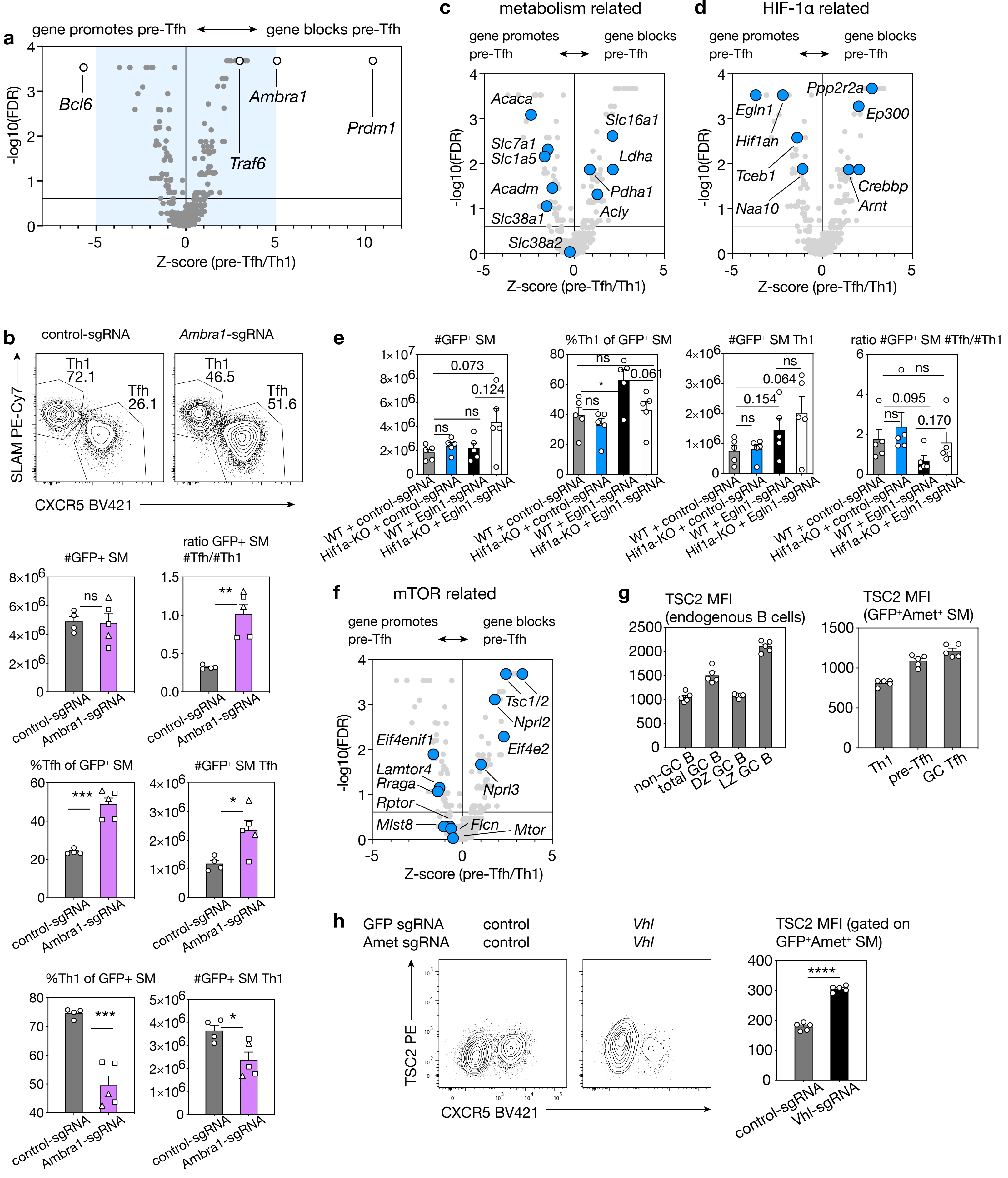
a
gene promotes pre-Tfh
gene blocks pre-Tfh
-log10(FDR)
Z-score (pre-Tfh/Th1)
Bcl6
Ambra1
Prdm1
Traf6
b
control-sgRNA
Ambra1-sgRNA
Th1
72.1
Tfh
26.1
Th1
46.5
Tfh
51.6
SLAM PE-Cy7
CXCR5 BV421
#GFP+ SM
ratio GFP+ SM #Tfh/#Th1
%Tfh of GFP+ SM
#GFP+ SM Tfh
%Th1 of GFP+ SM
#GFP+ SM Th1
ns
**
***
*
c
metabolism related
gene promotes pre-Tfh
gene blocks pre-Tfh
Acaca
Slc7a1
Slc1a5
Acadm
Slc38a1
Slc38a2
Slc16a1
Ldha
Pdha1
Acly
d
HIF-1α related
Egln1
Hif1an
Tceb1
Naa10
Ppp2r2a
Ep300
Crebbp
Arnt
e
#GFP+ SM
%Th1 of GFP+ SM
#GFP+ SM Th1
ratio #GFP+ SM #Tfh/#Th1
0.073
0.124
0.061
0.064
0.154
0.095
0.170
WT + control-sgRNA
Hif1a-KO + control-sgRNA
WT + Egln1-sgRNA
Hif1a-KO + Egln1-sgRNA
f
mTOR related
Tsc1/2
Nprl2
Eif4e2
Nprl3
Mtor
Flcn
Eif4enif1
Lamtor4
Rraga
Rptor
Mlst8
g
TSC2 MFI (endogenous B cells)
non-GC B
total GC B
DZ GC B
LZ GC B
TSC2 MFI (GFP+Amet+ SM)
Th1
pre-Tfh
GC Tfh
h
GFP sgRNA
Amet sgRNA
control
control
Vhl
Vhl
TSC2 PE
CXCR5 BV421
TSC2 MFI (gated on GFP+Amet+ SM)
****
control-sgRNA
Vhl-sgRNA

Supplementary Figure 7. Expanded screen hits and validation.

a, Hits from comparison of pre-Tfh and Th1 cells, in the expanded CRISPR screen described in Fig. 5a. **b**, Representative flow plots, Tfh and Th1 cells percentages and numbers, and ratio of GFP^+ SMARTA Tfh to Th1 cells, from $Cas9^+$ SMARTA cells transduced with control- or *Ambra1*-sgRNA, on day 6 post-LCMV infection, n=4 mice for control-sgRNA, 5 for *Ambra1*-sgRNA. For *Ambra1* sgRNAs, squares and triangles represent sgRNAs #1 and 2, respectively. **c**, **d** Gene hits related to metabolism (c) and HIF-1a (d) are highlighted in volcano plots comparing pre-Tfh and Th1 cells. **e**, Total GFP^+ SMARTA, percentage GFP^+ SMARTA Th1 cells, total GFP^+ SMARTA Th1 cells, and ratio of GFP^+ SMARTA Tfh to Th1 cells, from WT and Hif1a-KO $Cas9^+$ SMARTA cells transduced with the indicated sgRNAs, on day 6 post-LCMV infection, n=5 mice/group, for the experiment in Fig. 5d. **f,** Gene hits related to mTOR analyzed as in c-d. **g**, TSC2 staining in endogenous WT B cells and transferred $Cas9^+$ SMARTA cells transduced with control-sgRNA, on day 6 post-LCMV infection, n=5 mice/group. GC B cells were gated as $B220^+CD19^+Fas^+GL\text{-}7^+$ cells, and further sub-gated into dark zone ($CXCR4^+CD86^-$) and light zone ($CXCR4^-CD86^+$). SMARTA Th1, pre-Tfh, and GC Tfh were gated as in Supplementary Fig. 2a. **h**, TSC2 staining in $Cas9^+$ SMARTA cells transduced with control- or *Vhl*-sgRNA, on day 6 post-LCMV infection, n=5 mice/group. Data in (b), (e), (g) and (h) are presented as mean values +SEM. Representative data for (b, e, g, h) shown from 1 of at least 2 independent experiments. *p<0.05; **p<0.01, ***p<0.001, ****p<0.0001 as evaluated by two-tailed unpaired Student's t test (b, e, h). Source data are provided as a Source Data file and in Supplementary Data 8.

FIG S8

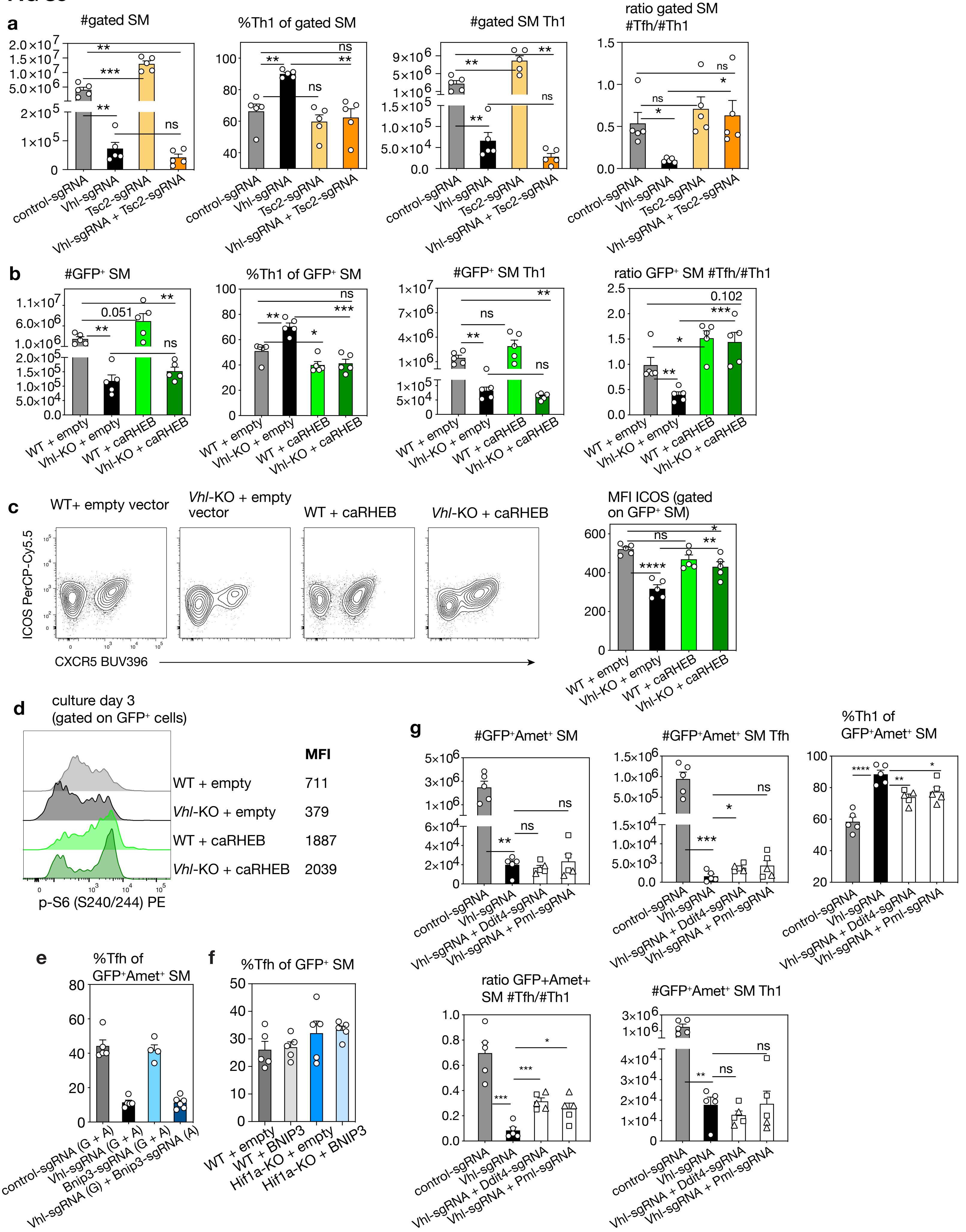

a
#gated SM
%Th1 of gated SM
#gated SM Th1
ratio gated SM #Tfh/#Th1
control-sgRNA
Vhl-sgRNA
Tsc2-sgRNA
Vhl-sgRNA + Tsc2-sgRNA
b
#GFP⁺ SM
%Th1 of GFP⁺ SM
#GFP⁺ SM Th1
ratio GFP⁺ SM #Tfh/#Th1
WT + empty
Vhl-KO + empty
WT + caRHEB
Vhl-KO + caRHEB
c
WT+ empty vector
Vhl-KO + empty vector
WT + caRHEB
Vhl-KO + caRHEB
ICOS PerCP-Cy5.5
CXCR5 BUV396
MFI ICOS (gated on GFP⁺ SM)
d
culture day 3 (gated on GFP⁺ cells)
MFI
WT + empty 711
Vhl-KO + empty 379
WT + caRHEB 1887
Vhl-KO + caRHEB 2039
p-S6 (S240/244) PE
e
%Tfh of GFP⁺Amet⁺ SM
control-sgRNA (G + A)
Vhl-sgRNA (G + A)
Bnip3-sgRNA (G + A)
Vhl-sgRNA (G) + Bnip3-sgRNA (A)
f
%Tfh of GFP⁺ SM
WT + empty
WT + BNIP3
Hif1a-KO + empty
Hif1a-KO + BNIP3
g
#GFP⁺Amet⁺ SM
#GFP⁺Amet⁺ SM Tfh
%Th1 of GFP⁺Amet⁺ SM
ratio GFP+Amet+ SM #Tfh/#Th1
#GFP⁺Amet⁺ SM Th1
control-sgRNA
Vhl-sgRNA
Vhl-sgRNA + Ddit4-sgRNA
Vhl-sgRNA + Pml-sgRNA

Supplementary Figure 8. Increased mTORC1 activity rescues Tfh differentiation in *Vhl*-deficient T cells

a, Total number of gated SMARTA cells, percentages and total numbers of gated Th1 cells, ratio of gated Tfh to Th1 cells, for the experiment in Fig. 6a. SMARTA cells were gated on $GFP^{+}Ametrine^{+}$, except for the *Tsc2*-sgRNA group, which was gated on GFP^{+}. **b**, Total number of GFP^{+} SMARTA cells, percentages and total number of GFP^{+} SMARTA Th1 cells, and ratio of GFP^{+} SMARTA Tfh to Th1 cells, from the experiment in Fig. 6b. **c,** Representative flow plots and quantification of ICOS staining for the experiment in Fig. 6b. **d**, Phospho-S6 staining in WT or *Vhl*-KO SMARTA cells transduced with empty or caRHEB-expressing vector and cultured *in vitro* for 3d. **e**, Tfh cells percentages from $Cas9^{+}$ SMARTA cells transduced with the indicated sgRNA vectors, on day 6 post-LCMV infection, n=5 mice for control- and *Vhl*-sgRNA, 4 for *Bnip3*-sgRNA, 6 for *Vhl*-sgRNA + *Bnip3*-sgRNA. **f**, Tfh cell percentages from WT or *Hif1a*-KO SMARTA cells transduced with empty or BNIP3-expressing vector, on day 6 post-LCMV infection, n=5 mice/group. **g**, Total numbers of $GFP^{+}Ametrine^{+}$ cells and $GFP^{+}Ametrine^{+}$ Tfh and Th1 cells from $Cas9^{+}$ SMARTA cells transduced with the indicated sgRNA vectors, on day 6 post-LCMV infection, n=5 mice/group, from the experiment in Fig. 6d. Data in (a)-(c), (e)-(g) are presented as mean values +SEM. Representative data for (a-g) shown from 1 of at least 2 independent experiments. *$p<0.05$; **$p<0.01$, ***$p<0.001$, ****$p<0.0001$ as evaluated by two-tailed unpaired Student's t test (a, b, c, g). Source data are provided as a Source Data file.

FIG S9

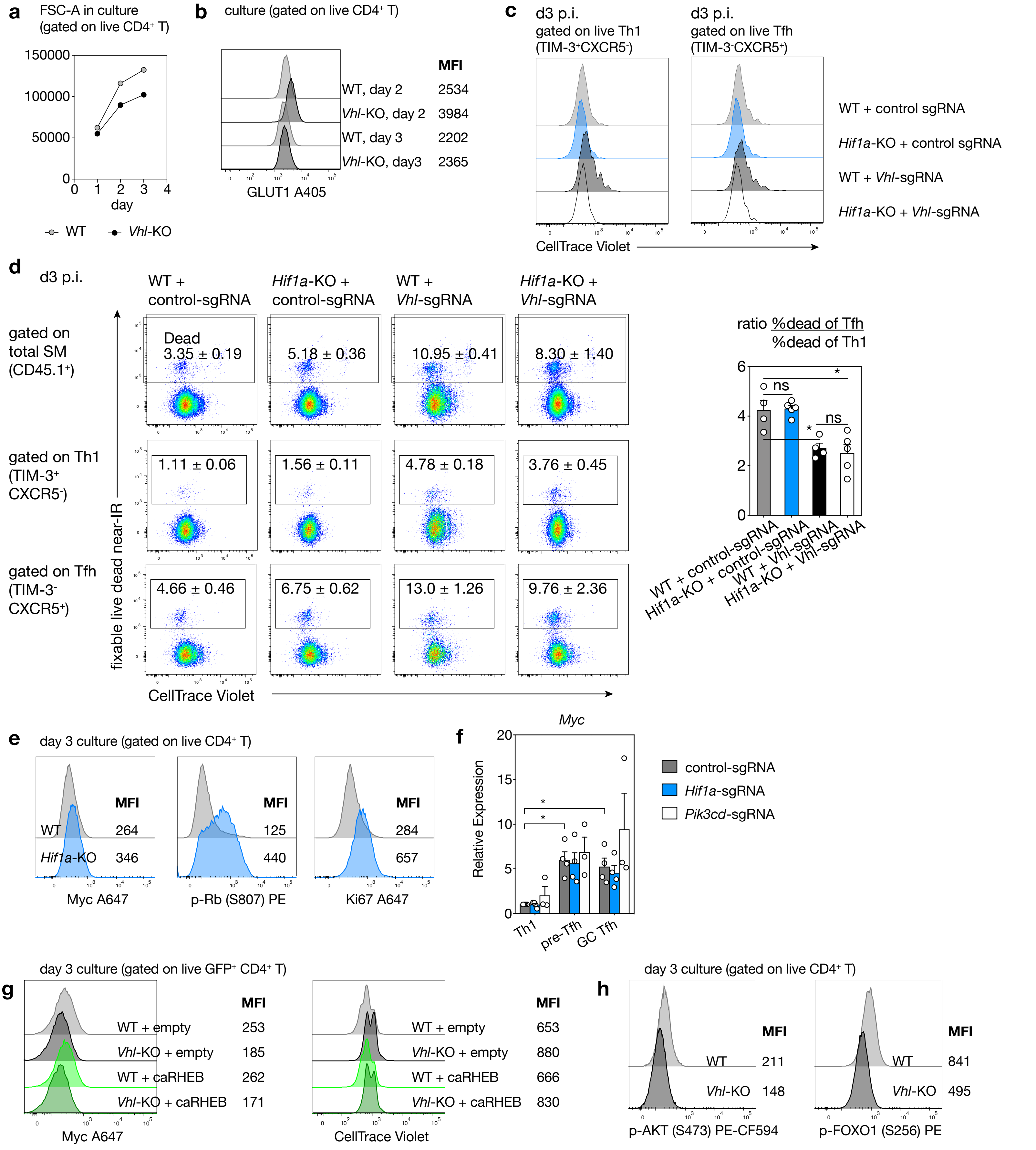
a
FSC-A in culture (gated on live CD4+ T)
150000
100000
50000
0
day
WT
Vhl-KO
b
culture (gated on live CD4+ T)
MFI
WT, day 2 2534
Vhl-KO, day 2 3984
WT, day 3 2202
Vhl-KO, day3 2365
GLUT1 A405
c
d3 p.i. gated on live Th1 (TIM-3+CXCR5-)
d3 p.i. gated on live Tfh (TIM-3-CXCR5+)
WT + control sgRNA
Hif1a-KO + control sgRNA
WT + Vhl-sgRNA
Hif1a-KO + Vhl-sgRNA
CellTrace Violet
d
d3 p.i.
WT + control-sgRNA
Hif1a-KO + control-sgRNA
WT + Vhl-sgRNA
Hif1a-KO + Vhl-sgRNA
gated on total SM (CD45.1+)
Dead
3.35 ± 0.19
5.18 ± 0.36
10.95 ± 0.41
8.30 ± 1.40
gated on Th1 (TIM-3+ CXCR5-)
1.11 ± 0.06
1.56 ± 0.11
4.78 ± 0.18
3.76 ± 0.45
gated on Tfh (TIM-3- CXCR5+)
4.66 ± 0.46
6.75 ± 0.62
13.0 ± 1.26
9.76 ± 2.36
fixable live dead near-IR
CellTrace Violet
ratio %dead of Tfh / %dead of Th1
ns
*
ns
*
WT + control-sgRNA
Hif1a-KO + control-sgRNA
WT + Vhl-sgRNA
Hif1a-KO + Vhl-sgRNA
e
day 3 culture (gated on live CD4+ T)
MFI
WT 264
Hif1a-KO 346
Myc A647
MFI
125
440
p-Rb (S807) PE
MFI
284
657
Ki67 A647
f
Myc
Relative Expression
Th1
pre-Tfh
GC Tfh
control-sgRNA
Hif1a-sgRNA
Pik3cd-sgRNA
g
day 3 culture (gated on live GFP+ CD4+ T)
MFI
WT + empty 253
Vhl-KO + empty 185
WT + caRHEB 262
Vhl-KO + caRHEB 171
Myc A647
MFI
WT + empty 653
Vhl-KO + empty 880
WT + caRHEB 666
Vhl-KO + caRHEB 830
CellTrace Violet
h
day 3 culture (gated on live CD4+ T)
MFI
WT 211
Vhl-KO 148
p-AKT (S473) PE-CF594
MFI
WT 841
Vhl-KO 495
p-FOXO1 (S256) PE

Supplementary Figure 9. Vhl is required for optimal Myc and cell proliferation.

a-**b**, WT or *Vhl*-KO SMARTA cells were cultured under Tfh-like conditions and evaluated for size by FSC (a) and GLUT1 expression (b). **c**, Representative CellTrace Violet dilution in Tfh and Th1 cells from WT or *Hif1a*-KO SMARTA cells transduced with control- or *Vhl*-sgRNA and transferred into WT hosts, day 3 post-LCMV infection. **d**, Representative viability dye staining in total SMARTA, Th1, or Tfh cells from WT or *Hif1a*-KO SMARTA cells transduced with control- or *Vhl*-sgRNA, day 3 post-LCMV infection, as in (c), n=4 mice for WT + control-sgRNA and WT + *Vhl*-sgRNA groups, 5 for *Hif1a*-KO + control-sgRNA and *Hif1a*-KO + *Vhl*-sgRNA /groups. Ratio of %dead among Tfh divided by %dead among Th1, for each group. **e**, WT or *Hif1a*-KO SMARTA cells were cultured under Tfh-like conditions, and stained as indicated on day 3. **f**, qRT-PCR measurement of *Myc* mRNA levels in sorted p.i. SMARTA cells, as described in Fig. 2c. **g**, WT or *Vhl*-KO SMARTA cells were stained with CellTrace Violet, transduced with empty or caRHEB-expressing vector at 24h post activation, and cultured *in vitro*, day 3. **h**, Phospho-AKT and phospho-FOXO1 staining in WT or *Vhl*-KO SMARTA cells cultured under Tfh-like conditions, day 3. Data in (d) and (f) are presented as mean values +SEM. Representative data for (a-e, g, h) shown from 1 of at least 2 independent experiments. *$p<0.05$ as evaluated by two-tailed unpaired Student's t test (d, f). Source data are provided as a Source Data file.

FIG S10

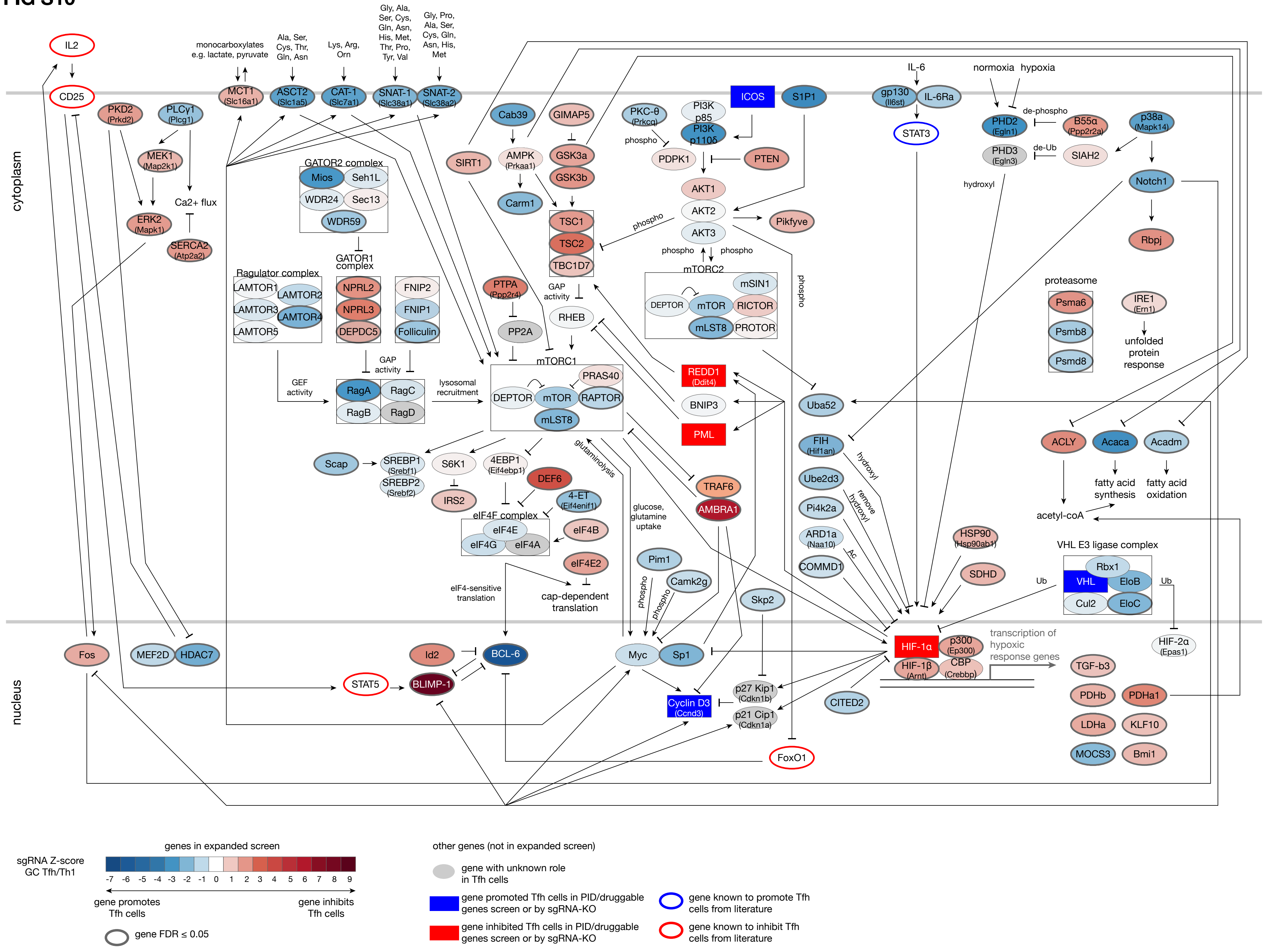

IL2
CD25
PKD2 (Prkd2)
PLCγ1 (Plcg1)
MEK1 (Map2k1)
ERK2 (Mapk1)
Ca2+ flux
SERCA2 (Atp2a2)
monocarboxylates e.g. lactate, pyruvate
Ala, Ser, Cys, Thr, Gln, Asn
Lys, Arg, Orn
Gly, Ala, Ser, Cys, Gln, Asn, His, Met, Thr, Pro, Tyr, Val
Gly, Pro, Ala, Ser, Cys, Gln, Asn, His, Met
MCT1 (Slc16a1)
ASCT2 (Slc1a5)
CAT-1 (Slc7a1)
SNAT-1 (Slc38a1)
SNAT-2 (Slc38a2)
cytoplasm
nucleus
GATOR2 complex
Mios
Seh1L
WDR24
Sec13
WDR59
GATOR1 complex
Ragulator complex
LAMTOR1
LAMTOR2
LAMTOR3
LAMTOR4
LAMTOR5
NPRL2
NPRL3
DEPDC5
FNIP2
FNIP1
Folliculin
GEF activity
GAP activity
RagA
RagC
RagB
RagD
lysosomal recruitment
Cab39
GIMAP5
AMPK (Prkaa1)
GSK3a
GSK3b
SIRT1
Carm1
TSC1
TSC2
TBC1D7
GAP activity
RHEB
PTPA (Ppp2r4)
PP2A
mTORC1
PRAS40
DEPTOR
mTOR
RAPTOR
mLST8
Scap
SREBP1 (Srebf1)
SREBP2 (Srebf2)
S6K1
IRS2
4EBP1 (Eif4ebp1)
DEF6
4-ET (Eif4enif1)
eIF4F complex
eIF4E
eIF4G
eIF4A
eIF4B
eIF4E2
eIF4-sensitive translation
cap-dependent translation
glutaminolysis
glucose, glutamine uptake
PKC-θ (Prkcq)
phospho
PI3K p85
PI3K p110δ
ICOS
S1P1
PDPK1
PTEN
AKT1
AKT2
AKT3
Pikfyve
phospho
phospho
phospho
mTORC2
mSIN1
DEPTOR
mTOR
RICTOR
mLST8
PROTOR
phospho
REDD1 (Ddit4)
BNIP3
PML
TRAF6
AMBRA1
Pim1
Camk2g
phospho
phospho
Skp2
Uba52
FIH (Hif1an)
Ube2d3
Pi4k2a
ARD1a (Naa10)
COMMD1
hydroxyl
remove hydroxyl
Ac
HSP90 (Hsp90ab1)
SDHD
IL-6
gp130 (Il6st)
IL-6Ra
STAT3
normoxia
hypoxia
PHD2 (Egln1)
de-phospho
B55α (Ppp2r2a)
PHD3 (Egln3)
de-Ub
SIAH2
hydroxyl
p38a (Mapk14)
Notch1
Rbpj
proteasome
Psma6
Psmb8
Psmd8
IRE1 (Ern1)
unfolded protein response
ACLY
Acaca
Acadm
fatty acid synthesis
fatty acid oxidation
acetyl-coA
VHL E3 ligase complex
Rbx1
VHL
EloB
Cul2
EloC
Ub
Ub
HIF-2α (Epas1)
HIF-1α
p300 (Ep300)
HIF-1β (Arnt)
CBP (Crebbp)
transcription of hypoxic response genes
TGF-b3
PDHb
PDHa1
LDHa
KLF10
MOCS3
Bmi1
Fos
MEF2D
HDAC7
STAT5
BLIMP-1
Id2
BCL-6
Myc
Sp1
Cyclin D3 (Ccnd3)
p27 Kip1 (Cdkn1b)
p21 Cip1 (Cdkn1a)
CITED2
FoxO1
genes in expanded screen
sgRNA Z-score GC Tfh/Th1
-7 -6 -5 -4 -3 -2 -1 0 1 2 3 4 5 6 7 8 9
gene promotes Tfh cells
gene inhibits Tfh cells
gene FDR ≤ 0.05
other genes (not in expanded screen)
gene with unknown role in Tfh cells
gene promoted Tfh cells in PID/druggable genes screen or by sgRNA-KO
gene inhibited Tfh cells in PID/druggable genes screen or by sgRNA-KO
gene known to promote Tfh cells from literature
gene known to inhibit Tfh cells from literature

Supplementary Figure 10. Expanded screen reveals feedback regulation of mTORC1 signaling through HIF-1a and Myc

Network map of signaling components in mTOR/HIF-1a pathways, including canonical genes and genes found to be statically significant hits (thick grey border) from the expanded CRISPR screen as described in Fig. 5a. Genes from the expanded screen are in ovals and Z-scores of GC Tfh versus Th1 enrichment is color-coded by shading as shown in the legend. Genes identified by the druggable screen or individual sgRNAs are indicated in blue or red boxes. Genes known from the literature are outlined in blue or red ovals.

FIG S11

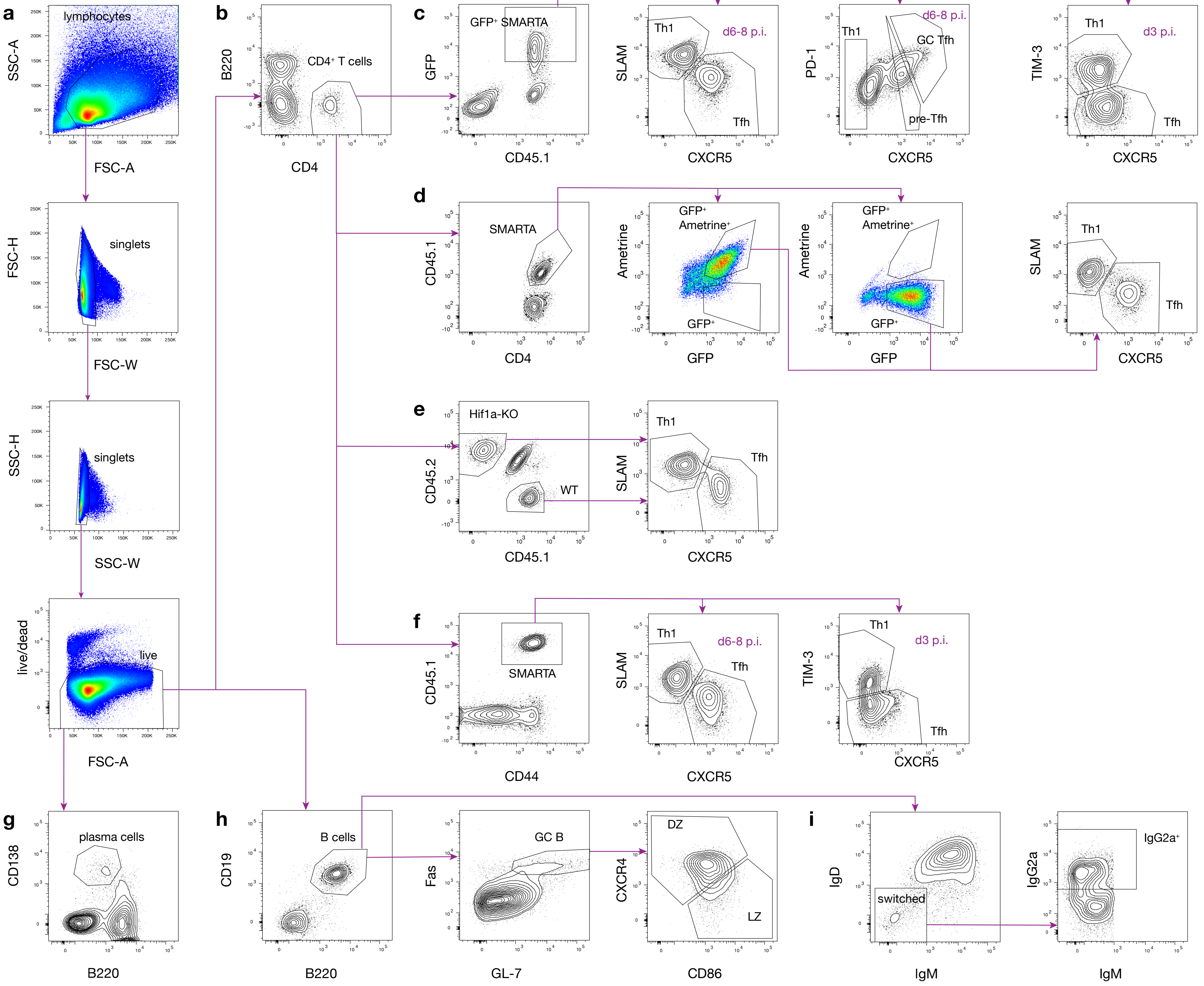
a
lymphocytes
SSC-A
FSC-A
singlets
FSC-H
FSC-W
singlets
SSC-H
SSC-W
live
live/dead
FSC-A
b
CD4+ T cells
B220
CD4
c
GFP+ SMARTA
GFP
CD45.1
Th1
d6-8 p.i.
Tfh
SLAM
CXCR5
Th1
d6-8 p.i.
GC Tfh
pre-Tfh
PD-1
CXCR5
Th1
d3 p.i.
Tfh
TIM-3
CXCR5
d
SMARTA
CD45.1
CD4
GFP+
Ametrine+
GFP+
Ametrine
GFP
GFP+
Ametrine+
GFP+
Ametrine
GFP
Th1
Tfh
SLAM
CXCR5
e
Hif1a-KO
WT
CD45.2
CD45.1
Th1
Tfh
SLAM
CXCR5
f
SMARTA
CD45.1
CD44
Th1
d6-8 p.i.
Tfh
SLAM
CXCR5
Th1
d3 p.i.
Tfh
TIM-3
CXCR5
g
plasma cells
CD138
B220
h
B cells
CD19
B220
GC B
Fas
GL-7
DZ
LZ
CXCR4
CD86
i
switched
IgD
IgM
IgG2a+
IgG2a
IgM

Supplementary Figure 11. Gating strategies for in vivo experiments

a, Gating strategy from total cells to live cells. The initial gating strategy included: FSC-A/SSC-A, exclusion of doublets (through FSC-H/FSC-W then SSC-H/SSC-W), and live cells (negative for Aqua or Blue fixable viability stain). These were followed by specific gating strategies. **b**, Gating strategy from live cells to CD4 T cells. **c**, Gating strategy from CD4 T cells to GFP^+ SMARTA cells, then Th1 and Tfh cells (by SLAM vs CXCR5, d6-8 post-infection), or Th1, pre-Tfh, and GC Tfh cells (by PD-1 vs CXCR5, d6-8 post-infection), or Tfh and Th1 cells (by TIM-3 vs CXCR5, d3 post-infection). Used for analysis in Fig 1c, 2b, 3c-d, 5d, 6b, Supplementary Fig. S1g-h, S1j-k, S2d-f, S4b, S4c, S4f, S5d-e, S6a, S7b, S7e, S8b, S8-c, S8f, S9c, S9d. Used for sort for Fig 1d-h (PID genes screen, SLAM vs CXCR5), Fig 2a, Supplementary Fig S2a-c (druggable gene targets screen, PD-1 vs CXCR5), Fig 2c, 4d-e, Supplementary Fig S5a-c, S9f (RT-PCR samples, PD-1 vs CXCR5), 5a-c, 5e, Supplementary Fig S7a, S7c, S7d, S7f, S10 (expanded screen, PD-1 vs CXCR5). Post-sort fractions had higher than 95% purify, as verified by flow cytometry analysis on the same FACSAria machine used to sort the cells. **d**, Gating strategy from CD4 T cells to $GFP^+Ametrine^+$ SMARTA cells and GFP^+ SMARTA cells, then Tfh and Th1 cells. $GFP^+Ametrine^+$ SMARTA cells gating used for Fig. 3e, 6a (control + control sgRNAs, *Vhl* + *Vhl* sgRNAs, and *Tsc2* + *Vhl* sgRNAs conditions), 6d, Supplementary Fig. S4d, S4e, S6b, S6c, S7g (right), S7h, S8a (control + control sgRNAs, *Vhl* + *Vhl* sgRNAs, and *Tsc2* + *Vhl* sgRNAs conditions), S8e, S8g. GFP^+ SMARTA cells gating used for Fig. 6a (*Tsc2*-sgRNA only condition) and Fig. S8a (*Tsc2*-sgRNA only condition). **e**, Gating strategy from live CD4 T cells to co-transferred WT and *Hif1a*-KO SMARTA cells, then Tfh and Th1 cells. Used for Fig. 2d-e. **f**, Gating strategy from live CD4 T cells to SMARTA cells, then Tfh and Th1 cells. Used for analysis in Fig. 3b, Supplementary Fig. S3b-e, S4a. Used for sort for Fig 4a-c, Supplementary Fig S5a, S5b (RNA-seq samples). **g**, Gating strategy from live cells to plasma cells. Used for Fig 2f. **h**, Gating strategy from live cells to B cells, to GC B cells, to DZ and LZ GC B cells, used for Fig. 2f, 3b, Supplementary Fig. S3f, S7g (left). **i**, Gating strategy from B cells to isotype-switched B cells, to IgG2a+ switched B cells. Used for Supplementary Fig. S3f.
