# Supplementary material for: In vivo CRISPR screens reveal a HIF-1α-mTOR-network regulates T follicular helper versus Th1 cells
Source: Nat Commun. 2022 Feb 10;13:805. doi: 10.1038/s41467-022-28378-6 (PMC8831505; doi:10.1038/s41467-022-28378-6)
Supplement: Supplementary file 2 — Description of additional Supplementary File [file 41467_2022_28378_MOESM2_ESM.pdf]

Descriptions of Additional Supplementary Files

Supplementary Data 1. Sequences of primers for cloning, fluorescent PCR, real-time PCR, CRISPR sgRNAs (Excel)

Supplementary Data 2. Sequences of CRISPR sgRNAs in primary immunodeficiency genes sgRNA library (Excel)

Supplementary Data 3. Normalized sgRNA read counts for samples from primary immunodeficiency genes sgRNA library screen (Excel)

Supplementary Data 4. Sequences of CRISPR sgRNAs in druggable target genes sgRNA library (Excel)

Supplementary Data 5. Normalized sgRNA read counts for samples from druggable target genes sgRNA library screen (Excel)

Supplementary Data 6. Differentially expressed genes from RNA-seq of *Hif1a*-KO versus WT SMARTA cells, post-LCMV infection, with p values adjusted for multiple testing (Excel)

Supplementary Data 7. Sequences of CRISPR sgRNA in expanded mTOR-HIF1a network genes sgRNA library (Excel)

Supplementary Data 8. Normalized sgRNA read counts for samples from expanded mTOR-HIF1a genes sgRNA library screen (Excel)

Supplementary Data 9. Antibodies and other flow cytometry reagents used in this study (Excel)
