# Supplementary material for: In vivo CRISPR screens reveal a HIF-1α-mTOR-network regulates T follicular helper versus Th1 cells
Source: Nat Commun. 2022 Feb 10;13:805. doi: 10.1038/s41467-022-28378-6 (PMC8831505; doi:10.1038/s41467-022-28378-6)
Supplement: Supplementary file 12 — Reporting Summary [file 41467_2022_28378_MOESM12_ESM.pdf]

nature research

Corresponding author(s): Pamela L. Schwartzberg, Bonnie Huang

Last updated by author(s): Dec 23, 2021

Reporting Summary

Nature Research wishes to improve the reproducibility of the work that we publish. This form provides structure for consistency and transparency in reporting. For further information on Nature Research policies, see our Editorial Policies and the Editorial Policy Checklist.

Statistics

For all statistical analyses, confirm that the following items are present in the figure legend, table legend, main text, or Methods section.

n/a	Confirmed	
☐	☒	The exact sample size (*n*) for each experimental group/condition, given as a discrete number and unit of measurement
☐	☒	A statement on whether measurements were taken from distinct samples or whether the same sample was measured repeatedly
☐	☒	The statistical test(s) used AND whether they are one- or two-sided<br>*Only common tests should be described solely by name; describe more complex techniques in the Methods section.*
☐	☒	A description of all covariates tested
☐	☒	A description of any assumptions or corrections, such as tests of normality and adjustment for multiple comparisons
☐	☒	A full description of the statistical parameters including central tendency (e.g. means) or other basic estimates (e.g. regression coefficient) AND variation (e.g. standard deviation) or associated estimates of uncertainty (e.g. confidence intervals)
☐	☒	For null hypothesis testing, the test statistic (e.g. *F*, *t*, *r*) with confidence intervals, effect sizes, degrees of freedom and *P* value noted<br>*Give P values as exact values whenever suitable.*
☒	☐	For Bayesian analysis, information on the choice of priors and Markov chain Monte Carlo settings
☒	☐	For hierarchical and complex designs, identification of the appropriate level for tests and full reporting of outcomes
☒	☐	Estimates of effect sizes (e.g. Cohen's *d*, Pearson's *r*), indicating how they were calculated

Our web collection on statistics for biologists contains articles on many of the points above.

Software and code

Policy information about availability of computer code

Data collection	Flow cytometry data was collected using BD FACSDiva v8.<br>CRISPR screen and RNA-seq experiments were performed using Illumina NextSeq System Suite v2.0 and samples were de-multiplexed using BaseSpace.<br>RT-PCR data was collected using QuantStudio 6.
Data analysis	Flow cytometry data was analyzed using FlowJo 10 (Treestar).<br>Statistical tests were performed in Prism 8 (Graphpad).<br>CRISPR screen reads were extracted from fastq files and normalized using custom perl scripts and Bowtie2. Custom scripts are available at https://lymphochip.nih.gov/local/CRISPR/ or upon request.<br>SgRNA abundance comparisons between samples were analyzed using Mageck 0.5.9.<br>RNA-seq data was analyzed by DESeq2 1.26 (part of Bioconductor 3.10) to find differentially expressed genes, and GSEA v4 to calculate gene set enrichment scores.

For manuscripts utilizing custom algorithms or software that are central to the research but not yet described in published literature, software must be made available to editors and reviewers. We strongly encourage code deposition in a community repository (e.g. GitHub). See the Nature Research guidelines for submitting code & software for further information.

Data

Policy information about availability of data

All manuscripts must include a data availability statement. This statement should provide the following information, where applicable:

- Accession codes, unique identifiers, or web links for publicly available datasets
- A list of figures that have associated raw data
- A description of any restrictions on data availability

The materials, data, and any associated protocols that support the findings of this study are available from the corresponding authors upon request.
The RNA-seq data is deposited in GEO under accession number GSE144467 (https://www.ncbi.nlm.nih.gov/geo/query/acc.cgi?acc=GSE144467).

Field-specific reporting

Please select the one below that is the best fit for your research. If you are not sure, read the appropriate sections before making your selection.

- [x] Life sciences
- [ ] Behavioural & social sciences
- [ ] Ecological, evolutionary & environmental sciences

For a reference copy of the document with all sections, see nature.com/documents/nr-reporting-summary-flat.pdf

Life sciences study design

All studies must disclose on these points even when the disclosure is negative.

Sample size	No statistical method was used to predetermine sample size. Sample size was based on previous and/or pilot studies, and was guided by the 3R principles to reduce animal numbers (Hubrecht, Animals 2019, PMID: 31575048). In most experiments, 4-6 mice were used per group.
Data exclusions	In Supplementary Data 3, Tfh and Tfr were not included from one mLN (out of 11 samples) due to poor staining quality.
Replication	Most of the experiments were reproduced at least 2 independent times with comparable results. Information on biological replicates is included in figure legends. Where representative data were shown, the experimental findings were reproduced with similar results. When data are pooled from multiple experiments, this is indicated in the figure legend.
Randomization	For baseline characterization experiments, animals were allocated into experimental groups based on genotype. For LCMV experiments, animals were allocated into groups depending on the genotype of adoptively transferred cells they received; all animals within an experiment were injected with cells at the same time, and infected with LCMV from the same stock at the same time.<br>For all animals experiments, animals were age and sex-matched, and littermates were used when possible. Either male or female mice were used.
Blinding	Flow cytometry experiments were not blinded due to limited personnel availability for experiments, but data were collected and analyzed as a group. CRISPR screen samples and RNA-seq samples were sequenced by a blinded co-author.

Reporting for specific materials, systems and methods

We require information from authors about some types of materials, experimental systems and methods used in many studies. Here, indicate whether each material, system or method listed is relevant to your study. If you are not sure if a list item applies to your research, read the appropriate section before selecting a response.

Materials & experimental systems

n/a	Involved in the study
[ ]	[x] Antibodies
[ ]	[x] Eukaryotic cell lines
[x]	[ ] Palaeontology and archaeology
[ ]	[x] Animals and other organisms
[x]	[ ] Human research participants
[x]	[ ] Clinical data
[x]	[ ] Dual use research of concern

Methods

n/a	Involved in the study
[x]	[ ] ChIP-seq
[ ]	[x] Flow cytometry
[x]	[ ] MRI-based neuroimaging

Antibodies

Antibodies used	Antibody, vendor, catalog #, clone, lot #, dilution<br><br>Flow Cytometry<br>anti-human PE secondary antibody, Jackson ImmunoResearch, 109-116-098, goat polyclonal, 135127, 1:100

anti-rabbit A647 secondary antibody, Thermo, A-21245, goat polyclonal, 205-1068, 1:1000
anti-rabbit BV421 secondary antibody, Biolegend, 406410, donkey polyclonal, B278806, 1:1000
B220 A700, Biolegend, 103231, RA3-6B2, 4335236, 1:300
Bcl-6 A647, BD, 561525, K112-91, 9176164, 1:20
Bcl-6 PE-CF594, BD, 562401, K112-91, 0223247, 1:20
Biotin-SP (long spacer) AffiniPure Fab Fragment Goat Anti-Rat IgG (H+L), Jackson ImmunoResearch, 112-067-003, goat polyclonal, 128191, 1:1000
Bnip3, Cell Signaling, 3769, rabbit polyclonal, 2, 1:100
CD138 BV605, Biolegend, B232065, 281-2, B240942, 1:300
CD4 PerCP-Cy5.5, Thermo, 45-0042-82, RM4-5, 1989136, 1:300
CD40L APC, Biolegend, 106510, MR1, B279299, 1:500
CD44 APC-Cy7, Biolegend, 103028, IM7, B262797, 1:200
CD45.1 PerCP-Cy5.5, Biolegend, 110728, A20, B252912, 1:200
CD45.2 APC, Thermo, 17-0454-82, 104, E07106-1631, 1:200
CXCR5, BD, 551961, 2G8, 6343832, 1:100
Fas BV421, BD, 562633, Jo2, 7047594, 1:100
Fixable Viability Dye Aqua, Thermo, L34966, N/A, 2069643, 1:200
Fixable Viability Dye Blue, Thermo, L34962, N/A, 2021206, 1:200
Fixable Viability Dye Near-IR, Thermo, L34976, N/A, 1985352, 1:1000
Foxp3 eF450, Thermo, 48-5773-82, FJK-16s, E10036-1634, 1:100
GL-7 A647, Biolegend, 144606, GL7, B207559, 1:400
Glut1 A405, abcam, ab210438, EPR3915, GR3271918-2, 1:500
Glut1 A647, abcam, ab195020, EPR3915, GR3207999-1, 1:1000
HIF-1a PE, Cell Signaling, 59370, D1S7W, 1, 1:50
ICOS PerCP-Cy5.5, Biolegend, 117424, 7E.17G9, B304595, 1:100
ICOS PE-Cy7, Thermo, 25-9942-82, 7E.17G9, E17665-103, 1:100
IL-21R/Fc, R&D, 596-MR, N/A, EFS0417031, 1:20
Ki67 A647, BD, 561126, B56, 3158729, 1:500
Myc A647, Cell Signaling, 13871, D84C12, 7, 1:200
p-Akt S473 PE-CF594, BD, 562465, M89-61, 728727, 1:50
p-FOXO1 S256, Cell Signaling, 9461S, rabbit polyclonal, 8, 1:200
p-Rb S807/S811 PE, Cell Signaling, 11917, D20B12, 4, 1:200
p-S6 S240/244 A647, Cell Signaling, 5044S, D68F8, 8, 1:1000
p-S6 S240/244 PE, Cell Signaling, 14236, D68F8, 3, 1:2000
PD-1 BV605, Biolegend, 135219, RMP-130, B244951, 1:200
PD-1 PE-Cy7, Biolegend, 109110, RMP-130, B218703, 1:100
PD-1 PE-Dazzle, Biolegend, 109116, RMP-130, B242949, 1:200
pimonidazole, Hypoxyprobe, hp12-100kit, rabbit polyclonal, 10-14-15, 1:200
SAv APC, BD, 554067, N/A, 7040924, 1:500
SAv BUV396, BD, 564176, N/A, 0113879, 1:100
SAv BV421, Biolegend, 405225, N/A, B282803, 1:500
SAv BV605, Biolegend, 405229, N/A, B239651, 1:200
SAv PE-Dazzle, Biolegend, 405247, N/A, B245596, 1:500
SLAM APC, Biolegend, 115910, TC15-12F12.2, B163400, 1:100
SLAM BV605, Biolegend, 115927, TC15-12F12.2, B265070, 1:100
SLAM BV650, Biolegend, 115932, TC15-12F12.2, B305945, 1:100
SLAM BV786, Biolegend, 115937, TC15-12F12.2, B283643, 1:100
SLAM PE-Cy7, Biolegend, 115914, TC15-12F12.2, B238925, 1:100
T-bet APC, Thermo, 17-5825-82, 4B10, 2015264, 1:200
T-bet PE-Cy7, Biolegend, 644824, 4B10, B290856, 1:200
TCF1, Cell Signaling, 2203S, C63D9, 6, 1:200
Thy1.1 APC, Biolegend, 202526, OX-7, B179776, 1:400
Thy1.2 PE, Biolegend, 140308, 53-2.1, B195369, 1:400
Tim3 APC, Biolegend, 119706, RMT3-23, B224474, 1:200
Tim3 PE-Cy7, Thermo, 25-5870-82, RMT3-23, 4342910, 1:100
TSC2, Cell Signaling, 4308, D93F12, 6, 1:100

Cell Culture
CD3e, Bioxcell, BE0001-1, 145-2C11, 5236/0215
CD28, Bioxcell, BE0015-1, 37.51, 4563/1112
IFN-g, Bioxcell, BE0055, XMG1.2, 5959/20116
IL-4, Bioxcell, BE0045, 11B11, 4513/0113
IL-12, Bioxcell, BE0051, C17.8, 4532/1112
TGF-b, Bioxcell, BE0057, 1D11.16.8, 509

Validation

All antibodies used in this study are from commercial sources and have been validated by the vendors. Validation data are available on the manufacturers' websites. Vendor descriptions for their validation processes are listed below:
https://www.abcam.com/primary-antibodies/how-we-validate-our-antibodies#Flow%20cytometry
https://www.biolegend.com/en-us/quality/product-development

https://www.bdbiosciences.com/en-us/products/reagents/flow-cytometry-reagents/research-reagents/quality-and-reproducibility
https://www.cellsignal.com/about-us/our-approach-process/antibody-validation-flow-cytometry
https://www.rndsystems.com/products/rd-systems-approach-antibody-quality
https://www.thermofisher.com/us/en/home/life-science/antibodies/invitrogen-antibody-validation.html

Eukaryotic cell lines

Policy information about cell lines

Cell line source(s)	293T cell line (ATCC)
Authentication	ATCC authenticated the 293T cell line by STR profiling. We did not further authenticate this line after purchase.
Mycoplasma contamination	The 293T cell line was not tested for mycoplasma.
Commonly misidentified lines (See ICLAC register)	No commonly misidentified cell lines were used in the study (according to ICLAC register v11).

Animals and other organisms

Policy information about studies involving animals; ARRIVE guidelines recommended for reporting animal research

Laboratory animals	All strains were on a C57BL6/J background.
Wild animals	No wild animals were used in this study.
Field-collected samples	No field-collected samples were used in this study.
Ethics oversight	Animal husbandry and experiments were performed in accordance with protocols approved by Animal Use and Care Committees of the National Human Genome Research Institute (NHGRI protocol G98.3) or National Institutes of Neurological Diseases and Stroke (NINDS protocol 1295-21), National Institutes of Health, Animal Welfare Assurance #A-4149-01.

Note that full information on the approval of the study protocol must also be provided in the manuscript.

Flow Cytometry

Plots

Confirm that:

- [x] The axis labels state the marker and fluorochrome used (e.g. CD4-FITC).
- [x] The axis scales are clearly visible. Include numbers along axes only for bottom left plot of group (a 'group' is an analysis of identical markers).
- [x] All plots are contour plots with outliers or pseudocolor plots.
- [x] A numerical value for number of cells or percentage (with statistics) is provided.

Methodology

Sample preparation	Spleen or peripheral lymph nodes were placed in nylon mesh cell strainers in FACS buffer (PBS + 0.5% BSA) and dissociated with a 3 ml syringe plunger to obtain single-cell suspensions. Spleen samples were resuspended in 1 ml ACK (Ammonium Chloride) lysis buffer and incubated for 2 min at room temperature, then quenched with 5 ml FACS buffer and filtered through 70 micron filter. Cells were washed once and resuspended in FACS buffer.
Instrument	Cells were analyzed on an LSR II or Fortessa (BD). Cells were sorted on a FACSAria (BD).
Software	Flow cytometry data was collected with FACSDiva v8 and analyzed with Flowjo v10.
Cell population abundance	Post-sort fractions had higher than 95% purify, as verified by flow cytometry analysis on the same FACSAria machine used to sort the cells.
Gating strategy	Initial gating strategy performed: FSC-A/SSC-A, exclusion of doublets (through FSC-H/FSC-W then SSC-H/SSC-W), live cells (negative for Aqua or Blue fixable viability stain). These were followed by specific gating strategy reported in the figure legends of main and supplementary figures.

- [x] Tick this box to confirm that a figure exemplifying the gating strategy is provided in the Supplementary Information.
